# The BRAF V600E/MEK/ERK/METTL3 positive feedback loop regulates autophagy and promotes stemness and invasiveness in glioblastoma via m^6^A modification

**DOI:** 10.1186/s13046-025-03623-0

**Published:** 2025-12-30

**Authors:** Yuan Xie, Yan Li, Meiqin Tang, Zhi Li, Wanming Hu, Xiaoling Qiu, Changyu Wang, Yunzhi Zou, Jie Lu, Ze Yuan, Furong Chen, Yuanzhong Yang, Chen Lu, Ke Sai, Ying Guo, Zhenqiang He, Hao Duan, Yonggao Mou

**Affiliations:** 1 Department of Neurosurgery/Neuro-Oncology, State Key Laboratory of Oncology in South China, Guangdong Provincial Clinical Research Center for Cancer, Sun Yat-sen University Cancer Center, Guangzhou, China; 2 Department of Oncology, The First Affiliated Hospital of Guangzhou University of Chinese Medicine, Guangzhou, China; 3 Postdoctoral Research Station of Guangzhou University of Chinese Medicine, Guangzhou, China; 4 Department of Pathology, State Key Laboratory of Oncology in South China, Guangdong Provincial Clinical Research Center for Cancer, Sun Yat-sen University Cancer Center, Guangzhou, China; 5 Department of Clinical Research, State Key Laboratory of Oncology in South China, Guangdong Provincial Clinical Research Center for Cancer, Sun Yat-sen University Cancer Center, Guangzhou, China

**Keywords:** Glioblastoma, BRAF V600E, METTL3, Positive feedback loop, N6-methyladenosine

## Abstract

**Background:**

Epithelioid glioblastoma (GBM) is characterized by its highly aggressive behavior and the presence of the BRAF V600E mutation. However, the impact and underlying mechanisms of the BRAF V600E mutation on GBM stemness and invasiveness are unknown. Dysregulation of N6-methyladenosine (m^6^A) modification is closely associated with the progression of various cancers. The role of m^6^A modification in BRAF V600E-mutant GBM has not been defined.

**Methods:**

Functional assays were performed to evaluate the impact of BRAF V600E on stemness phenotypes of glioma stem-like cells (GSCs) and invasive phenotypes of GBM cells in vitro and in vivo. Mechanistic investigations involved m^6^A quantification, qPCR, western blotting, co-immunoprecipitation, MeRIP-seq, luciferase reporter assays, and transmission electron microscopy to elucidate the mechanism by which BRAF V600E regulates stemness and invasiveness. These findings were further supported by evidence from public GBM patient databases and tumor samples.

**Results:**

We found that BRAF V600E significantly upregulated METTL3 expression via ERK signaling in GSCs and GBM cells, thereby promoting stemness and invasiveness. METTL3 established a positive feedback loop with BRAF V600E to facilitate m^6^A modification enrichment, thereby inducing autophagy. The BRAF V600E-driving stemness and invasiveness were autophagy-dependent. In vivo experiments showed that BRAF V600E-expressing GBM was responsive to both the METTL3 inhibitor STM2457 and the autophagy inhibitor HCQ.

**Conclusions:**

This study reveals that the BRAF V600E/MEK/ERK/METTL3 positive feedback loop promotes autophagy, driving the stemness of GSCs and the invasiveness of GBM cells via m^6^A modification in vitro and in vivo. Our results suggest that METTL3 and autophagy are promising therapeutic targets in BRAF V600E-mutant GBM.

**Supplementary Information:**

The online version contains supplementary material available at 10.1186/s13046-025-03623-0.

## Background

Glioblastoma (GBM) is the most common and aggressive primary malignant brain tumor in adults, with a median overall survival of less than 2 years even after the standard therapy [1, 2]. The treatment responses across patients with diverse genotypes of GBM are constrained by high tumor heterogeneity driven by its complex molecular profile [3–5]. Therefore, elucidating the molecular drivers underpinning GBM is imperative to develop novel therapeutic approaches to improve patient outcomes.

BRAF is a key regulator of the RAF/MEK/ERK signaling cascade that governs cellular proliferation, differentiation, and survival [6]. The V600E mutation in BRAF confers constitutive kinase activity, leading to persistent activation of the downstream ERK signaling pathway, resulting in uncontrolled proliferation and enhanced invasiveness [7, 8]. In adult glioma patients, the prevalence of the BRAF V600E mutation is less than 5% [9]. However, in epithelioid glioblastoma (eGBM), the highly aggressive histological subtype of GBM, the prevalence approaches 70%, making it the glioma subtype with the highest prevalence of BRAF V600E mutation [9]. Patients with eGBM exhibit shorter survival compared with those with other GBM subtypes [10], suggesting that BRAF V600E is an adverse factor for prognosis. An integrated proteogenomic study revealed a strong association between BRAF kinase activity and enhanced tumor invasiveness and recurrence in GBM [11]. However, the regulatory network linking BRAF V600E to the malignant progression of GBM remains unclear. Elucidating this mechanism is crucial for achieving precision therapy in patients.

N6-methyladenosine, m^6^A, is one of the most prevalent and abundant RNA modifications [12, 13]. Studies have demonstrated that dysregulation of m^6^A modifications is closely linked to the tumorigenesis and progression of various cancers [14]. m^6^A is dynamically and reversibly regulated by two key catalytic proteins, methyltransferases (writers) and demethylases (erasers), and is recognized by m^6^A-binding proteins (readers) to mediate downstream signaling [15, 16]. As a crucial m^6^A methyltransferase, METTL3 has been shown to play a pivotal role in regulating malignant biological phenotypes such as cell proliferation, migration, stemness maintenance, and drug resistance by influencing the transcription, translation, and stability of various target RNAs in GBM and other malignancies [17–19]. Previous research suggested that m^6^A modification-mediated selective translational reprogramming contributes to acquired resistance to BRAF inhibitors in BRAF V600E-mutant melanoma, and METTL3 knockdown significantly enhanced the sensitivity of these melanoma cells to BRAF inhibitor treatment [20]. However, the relationship between m^6^A methylation and BRAF V600E in GBM remains unclear.

GBM contains glioma stem-like cells (GSCs), which drive the heterogeneity of GBM [21]. Characterized by tumor initiation, self-renewal, and sustained proliferation, GSCs can adapt to changes in the microenvironment and therapeutic stimuli, playing a critical role in GBM recurrence and treatment resistance [21–23]. Patient-derived xenograft (PDX) models established using patient-derived GSCs recapitulate tumor progression and treatment responses [24]. Multiple studies have suggested that autophagy activation maintains the stemness of various cancer stem cells, including GSCs, promotes tumor growth and treatment resistance [25–27]. While brain tumors with the BRAF V600E mutation exhibit autophagy dependency [28], the underlying mechanism remains unelucidated. These findings underscore the necessity of investigating the role of BRAF V600E in maintaining GSC stemness and its regulatory mechanisms governing autophagy.

Our study identified a BRAF V600E/METTL3 positive feedback loop that regulates autophagy through m^6^A modification, thereby influencing invasiveness and stemness of GBM. These findings provide preclinical evidence for targeting METTL3 or autophagy as a therapeutic strategy for BRAF V600E-mutant GBM.

## Materials and methods

### GBM patient samples

Surgical samples were obtained from patients undergoing resection for GBM at the Sun Yat-sen University Cancer Center. The Medical Ethics Committee of Sun Yat-sen University Cancer Centre approved this study (B2025-836-01). All procedures involving human participants were performed in accordance with the Declaration of Helsinki and its later amendments.

### Cell culture and treatments

Human GBM cell lines U87MG and U251 cells were obtained from the State Key Laboratory of Oncology in South China (Guangzhou, China). U87MG and U251 cells were grown in DMEM (C11995500BT, Gibco, USA) supplemented with 10% fetal bovine serum (FCS500, ExCell Bio, Suzhou, China) and 1% penicillin-streptomycin (ES-8440, EcoTop Bio, Guangzhou, China). Human GSCs (GSC1 and GSC2) were derived from fresh GBM patient samples as previously described [29–31]. GBM patient-derived GSC0147 were gifted from Dr. Nu Zhang (The First Affiliated Hospital of Sun Yat-sen University). GSCs were cultured in serum-free DMEM/F12 medium (C11330500BT, Gibco) supplemented with 2% B27 (12587010, Gibco), 20 ng/mL basic fibroblast growth factor (bFGF, 100-18B, Pepro Tech), and 20 ng/mL epidermal growth factor (EGF, AF-100-15, Pepro Tech). Cells were maintained under a humidified environment at 37 °C under 5% CO_2_.

Depending on the experimental purpose, cells were treated with 20 µM STM2457 (HY-134836, MedChem Express, USA) for 48 h (GBM cells) or 7 days (GSCs), 5 µg/mL hydroxychloroquine (HCQ, HY-B1370, MedChem Express) for 48 h (GBM cells) or 7 days (GSCs), 5 µg/mL actinomycin D (HY-17559, MedChem Express) for 0, 6, and 12 h, or 50 µg/mL cycloheximide (CHX, HY-12320, MedChem Express) for 0, 4, 8 and 12 h.

### CCK8 assay

GBM cells or GSCs (1000 cells/well) were seeded in 96-well plates and allowed to adhere. Cells were treated following the experimental conditions. Proliferation viability was assessed using the CCK8 reagent (C6005, NCM, Suzhou, China) by measuring absorbance at 450 nm. To determine the half-maximal inhibitory concentration (IC50) of STM2457, OD values were measured across a concentration gradient ranging from 0 to 200 µM.

### Live/dead cell assay

Cell suspensions were prepared and stained with Calcein-AM and Ethidium Homodimer-1 from the LIVE/DEAD Viability Kit (L3224, ThermoFisher, USA), following the manufacturer’s protocol. Cells were then subjected to assessment by a flow cytometer (CytoFLEX, Beckman Coulter, USA).

### Transwell assay

GBM cells (5 × 10⁴) in serum-free medium were seeded into upper chambers of Transwell chambers (353097, Falcon, USA); the lower chambers contained complete medium with 10% fetal bovine serum. After incubation for the indicated times, migrated cells were stained with crystal violet and imaged under a microscope. Stained cells were quantified using ImageJ (v1.53).

### Apoptosis assay

Cells were treated with HCQ or solvent for 48 h. Apoptosis was evaluated using the Annexin V-FITC/PI Apoptosis Kit (E-CK-A211, Elabscience, Wuhan, China) following the manufacturer’s instructions.

### SiRNA transfection and generation of stable cell lines

Cells were transiently transfected with BRAF siRNAs or negative control siRNA (Gene Pharma, Shanghai, China) using Lipofectamine 3000 (L3000015, ThermoFisher) following the manufacturer’s instructions. Stable cell lines were constructed using lentivirus encoding BRAF V600E (V600E), shMETTL3, and overexpressing METTL3 (METTL3 OE), luciferase, and the corresponding negative controls. The lentiviruses were generated by Genechem (Shanghai, China). The siRNA and shRNA sequences are shown in Supplementary Table S1.

### Sphere formation assay

GSCs from each experimental group were plated in 96-well plates at a density of 1 cell per 4 µL medium, with three technical replicates per group. After 7 days of culture, tumor spheres were counted for quantitative comparison.

### In vitro limiting dilution assay

For the limiting dilution assay, graded numbers of GSCs (1, 5, 10, 20, 30, 40 cells per well) were dispensed into 96-well plates, with eight replicate wells for each cell dose. Sphere formation was assessed on day 7. The self-renewal capacity of GSCs in each group was analyzed by the Extreme Limiting Dilution Analysis (ELDA) [32].

### Quantitative real-time PCR (qPCR)

Total RNA was extracted using an RNA Purification Kit (RN001, Yishan, Shanghai, China). The RNA was reverse-transcribed into cDNA with a reverse transcription kit (11141ES60, Yeasen, Shanghai, China). Real-time PCR was performed using Master qPCR Mix (TSE201, TSINGKE, Beijing, China). The relative expression levels of each gene were calculated using the 2^−ΔΔCT^ method and normalized using 18 s expression. The primer sequences are presented in Supplementary Table S2.

### m^6^A dot blot

RNA (100 ng) was heat-denatured at 95 °C for 3 min followed by immediate chilling on ice. Denatured RNA was spotted onto Hybond-N + membranes, and UV-crosslinked. Membranes were blocked with 5% non-fat milk and incubated overnight at 4 °C with m^6^A antibody (1:1000, 202 003, SYSY, Germany). Membranes were then incubated with secondary antibody (1:20000, ab6721, Abcam, UK). Signals were developed using enhanced chemiluminescence kit (K1231, APExBIO, USA). Membranes were stained with methylene blue to detect total RNA as loading control. Spot signal intensity was quantified using ImageJ.

### Western blot

Cells were lysed with RIPA lysis buffer (P0013C, Beyotime, Shanghai, China). Protein samples were separated on an SDS-PAGE gel and transferred onto a PVDF membrane. The membranes were incubated overnight at 4 °C with primary antibodies: anti-BRAF (1:1000, sc-5284, Santa Cruz, USA), anti-BRAF V600E (1:1000, MA5-24661, ThermoFisher), anti-SOX2 (1:1000, ab97959, Abcam), anti-Nestin (1:1000, MAB1259, R&D Systems, USA), anti-GFAP (1:1000, ab278054, Abcam) anti-METTL3 (1:1000, 86132, CST, USA), anti-ERK (1:1000, 9102, CST), anti-phospho-ERK (1:1000, 4370, CST), anti-MEK (1:1000, 8727, CST), anti-phospho-MEK (1:1000, 9154, CST), anti-LC3 (1:1000, 83506, CST), anti-SQSTM1 (1:1000, 88588, CST), anti-GAPDH (1:10000, TA802519, OriGene, USA) and anti-Tubulin (1:10000, TA506605, OriGene). The membranes were then incubated for 1 h with secondary antibodies (1:20000, ab6789 or ab6721, Abcam). Protein bands were detected using an enhanced chemiluminescence kit (K1231, APExBIO).

### Immunofluorescence staining

Cells were fixed, permeabilized, and blocked. Samples were incubated with primary antibodies against Nestin (MAB1259, R&D Systems), SOX2 (ab97959, Abcam), or CD133 (ab19898, Abcam) overnight at 4 °C, followed by incubation with fluorescently labeled secondary antibodies. Nuclei were counterstained with DAPI. Images were captured using a fluorescence microscope.

### Co-immunoprecipitation (Co-IP) and Immunoblotting

To assess whether p-ERK interacts with METTL3, GSCs and GBM cells were lysed in RIPA buffer supplemented with protease and phosphatase inhibitors. Equal amounts of protein were incubated with an anti-phospho-ERK antibody (4370, CST) or IgG Isotype Control (98136-1-RR, Proteintech) at 4 °C overnight, followed by capture with Protein A/G agarose beads for 2 h. After several washes with cold lysis buffer, bound proteins were released by boiling in SDS sample buffer. The precipitated samples and input lysates were separated by SDS-PAGE and transferred to PVDF membranes. METTL3 and p-ERK levels were examined using standard immunoblotting with an anti-METTL3 (86132, CST) and anti-phospho-ERK (4370, CST) antibody.

### Molecular dynamics simulation

The METTL3 and ERK2 structures were obtained from AlphaFold3, with ERK2 phosphorylated at Thr185/Tyr187 using MOE2019, followed by energy minimization. Docking was performed with HDOCKv1.1. The complex was solvated in a TIP3P water box (15 × 15 × 15 nm³, 1.2 nm buffer) and neutralized with ions using GROMACS 2022. Simulations utilized the Amber99sb-ildn force field, PME electrostatics (10 Å cutoff), and a 100 ns production run under NPT conditions (300 K, 1 bar) after NVT/NPT equilibration. Trajectories were analyzed for the root mean square deviation (RMSD), the root mean square fluctuation (RMSF), and the radius of gyration (Rg) with GROMACS tools.

### Methylated RNA Immunoprecipitation sequencing (MeRIP-seq) and RNA sequencing (RNA-seq)

After total RNA was extracted, the RNA was divided into two portions: one served as the input and the other was enriched using m^6^A -specific antibodies. After constructing sequencing libraries, MeRIP-seq and RNA-seq were performed by Gene Denovo Biotechnology (Guangzhou, China). For bioinformatics analysis, MACS2 (v2.1.2) was used to identify peaks. Motif analysis was performed on peak-associated transcripts using MEME Suite and DREME. Peaks with log2|FC|≥1 and FDR ≤ 0.05 were defined as significantly differentially methylated using DiffBind (v2.8). Gene expression abundance was quantified using the RSEM software. Functional annotation of differentially methylated peak-associated genes was performed by Kyoto encyclopedia of genes and genomes (KEGG) enrichment analyses. The Integrative Genomics Viewer was used to visualize genome-wide peak distributions.

### Luciferase reporter assay

The specific 3′ untranslated region (3′ UTR) regions of BRAF and SQSTM1 were cloned into the firefly luciferase reporter vectors. For BRAF, a mutation was introduced at the m^6^A methylation site (AAACA mutated to AATCA) using site-directed mutagenesis. In SQSTM1, the methylation site AGACC was mutated to AGTCC. Cells were transfected with reporter plasmids, and luciferase activity was quantified using the Dual Glo Luciferase Reporter Gene Assay Kit (11405ES60, Yeasen). Renilla luciferase activity was normalized to firefly luciferase activity to assess transfection efficiency. Sequences of wild-type and mutant m^6^A methylation sites are detailed in Supplementary Table S3.

### Transmission electron microscopy (TEM)

Cells were centrifuged, resuspended in TEM fixative, and embedded in 1% agarose. Cells were fixed with 1% OsO_4_ for 2 h. After dehydrating, samples were embedded with pure EMBed 812 overnight and polymerized at 60 °C for 48 h. The samples were cut into 60 nm sections, which were then stained with uranyl acetate and lead citrate. Sections were analyzed on a transmission electron microscope (HITACHI, HT7800, Japan) and images were captured.

### In vivo experiment

Animal experiments were approved by The Experimental Animal Ethics Committee of Sun Yat-sen University Cancer Center (L102012024100J). Male BALB/C nude mice (5 weeks old) and Male NOD-SCID mice (5 weeks old) purchased from Gempharmatech-GD (Guangzhou, China) were housed under specific pathogen-free conditions. For cell-derived tumor xenografts (CDX) models, BRAF wild-type or BRAF V600E-expressing U87MG cells (5 × 10^5^) were injected into the right brain of BALB/C nude mice. For patient-derived tumor xenografts (PDX) models, BRAF wild-type or BRAF V600E-expressing GSC1 cells (1 × 10^4^) were injected into the right brain of NOD-SCID mice. For orthotopic GBM modeling, cells were injected into the brain, 2 mm from the right of the bregma and 4 mm beneath the calvarial surface. Mice were intraperitoneally injected with PBS, STM2457 (50 mg/kg) or HCQ (50 mg/kg) every 2 days for a total of five doses. Bioluminescence images of tumors were captured using an in vivo imaging system (IVIS, Caliper Life Sciences, USA) and analyzed with Living Image software (Caliper Life Sciences) to quantify bioluminescence activity. On day 14 post-treatment, mice were sacrificed, and the brains were harvested and preserved as formalin-fixed, paraffin-embedded samples. Survival data were recorded for all experimental groups.

### Histological and immunohistochemical (IHC) analyses

Formalin-fixed, paraffin-embedded sections were deparaffinized, rehydrated, and subjected to hematoxylin-eosin (HE) or IHC staining. For IHC staining, after antigen retrieval and 3% H_2_O_2_ incubation, sections were blocked with 5% goat serum for 1 h and then incubated overnight at 4 °C with antibodies against BRAF V600E (1:200, MA5-24661, ThermoFisher), METTL3 (1:200, 86132, CST), Phospho-ERK (1:200, 4370, CST), SQSTM1 (1:400, 88588, CST), or Ki-67 (1:500, GB111499, Servicebio, Wuhan, China), followed by incubation with secondary antibody (10506001030, Panovue, Beijing, China). Signal detection was performed using 3,3-diaminobenzidine. The sections were counterstained using hematoxylin.

### Databases

The transcriptional data and gene expression correlations from GBM patients were obtained from public databases: The Cancer Genome Atlas (TCGA) via the cBioPortal for Cancer Genomics (http://www.cbioportal.org) and GEPIA 2 (http://gepia2.cancer-pku.cn), and the Chinese Glioma Genome Atlas (CGGA) (http://www.cgga.org.cn). The median BRAF expression of all patients was defined as the cut-off point for high and low expression.

### Statistical analysis

Statistical analyses were performed using GraphPad Prism 9. Data are expressed as the mean ± standard deviation (SD). All experiments were a minimum of three independent replications. Student’s t-test was used for two group comparisons. Survival differences were assessed using the log-rank test. *p* < 0.05 indicated statistical significance.

## Results

### BRAF V600E promotes the malignant phenotypes of GSCs and GBM cells

**Fig. 1:**
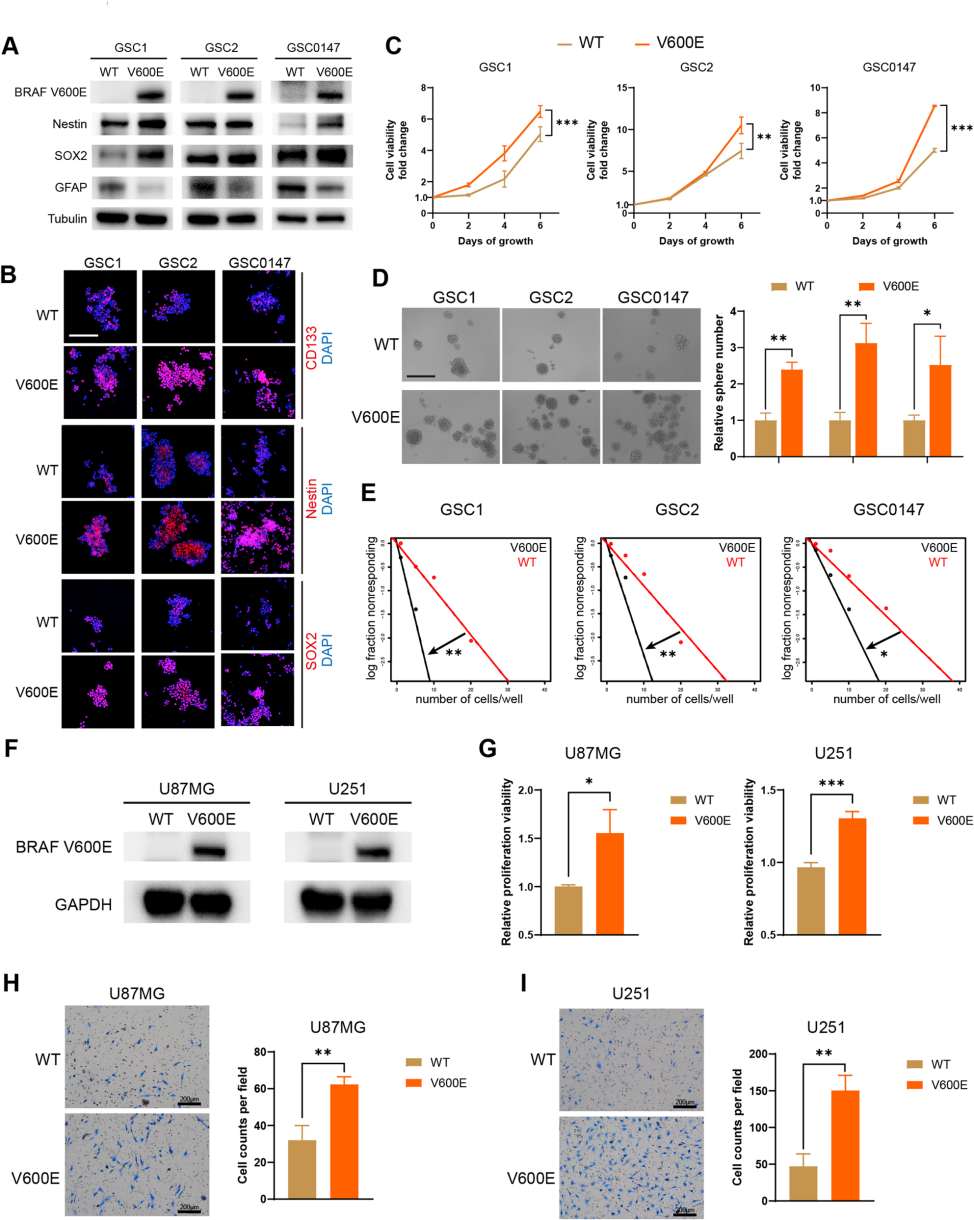
BRAF V600E promotes the malignant phenotypes of GSCs and GBM cells. **A** Western blot analysis of BRAF V600E, stemness-related markers (Nestin, SOX2), and the differentiation marker (GFAP) protein expression in GSCs with or without BRAF V600E. **B** Representative immunofluorescence images for the stemness markers Nestin, SOX2, and CD133 in GSCs with or without BRAF V600E. Nuclei are labeled with DAPI. Scale bar: 200 μm. **C** Cell viability of BRAF V600E-expressing GSCs compared with wild-type controls, assessed by CCK-8 assay. **D** Sphere formation of BRAF V600E-expressing GSCs compared with wild-type controls and quantification of relative sphere numbers. Scale bar: 400 μm. **E** In vitro limiting dilution assay of BRAF V600E-expressing GSCs compared with wild-type controls. **F** Validation of BRAF V600E protein expression in GBM cells after stable transfection with lentiviral vectors, as analyzed by western blot. **G** Proliferation viability in BRAF V600E-expressing GBM cells, assessed by CCK-8 assay. **H–I** Transwell assays assessing cell migration in wild-type or BRAF V600E-expressing GBM cells. Scale bars: 200 μm. WT, BRAF wild type; V600E, BRAF V600E. *p < 0.05, **p < 0.01, ***p < 0.001

The BRAF V600E mutant (hereafter referred to as BRAF V600E or V600E) exhibits the highest kinase activity among all BRAF kinase isoforms [8]. To investigate the role of BRAF V600E in GSCs, we expressed BRAF V600E in GSC1, GSC2, and GSC0147 using lentiviral transfection. Western blot confirmed the successful establishment of BRAF V600E-expressing GSCs and revealed that it upregulated the protein levels of stemness-related markers (Nestin and SOX2), along with a decrease in the differentiation marker GFAP (Fig. 1A). Immunofluorescence staining showed enhanced fluorescence intensity of stemness-related markers (CD133, Nestin, and SOX2) in BRAF V600E-expressing GSCs compared with the wild-type group, further supporting the stemness-promoting role of BRAF V600E (Fig. 1B). Stemness-related phenotypes confirmed the tumor-promoting effects of BRAF V600E. CCK-8 assays indicated that BRAF V600E significantly enhanced the proliferative activity of GSCs (Fig. 1C), while it also promoted the formation of tumor spheres according to sphere formation assays (Fig. 1D). Moreover, the in vitro limiting dilution assay demonstrated that BRAF V600E markedly increased the self-renewal capacity of GSCs (Fig. 1E).

Similarly, we expressed BRAF V600E in U87MG and U251 cells (BRAF wild-type GBM cell lines [33]) to investigate the role of BRAF V600E in GBM cells (Fig. 1F). CCK8 assays demonstrated that BRAF V600E significantly enhanced the viability of GBM cells (Fig. 1G). Transwell assays revealed a significant increase in the number of migratory GBM cells in the BRAF V600E-expressing group, and BRAF V600E-expressing GBM cells exhibited a typical migratory phenotype with more pronounced lamellipodia extension (Fig. 1H, I). These findings indicate that BRAF V600E exacerbates the malignant phenotypes of GBM cells.

### BRAF V600E facilitates m^6^A methylation enrichment by regulating METTL3 protein expression and stability

**Fig. 2:**
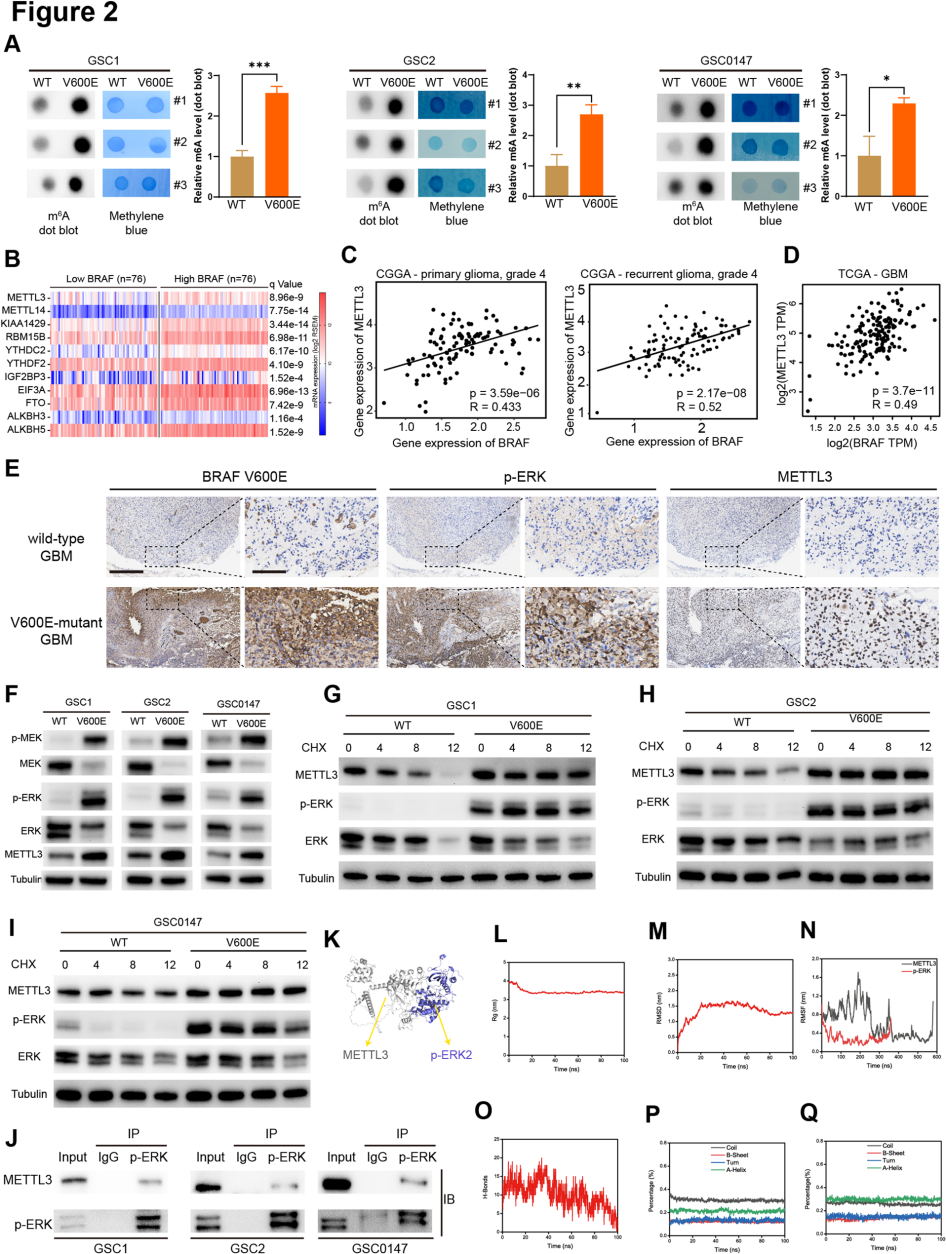
BRAF V600E facilitates m^6^A methylation enrichment by regulating METTL3 protein expression and stability. **A** m^6^A dot blot and quantification of global m^6^A abundance in GSCs with or without BRAF V600E. **B** Analysis of m^6^A writer, eraser, and reader expressions in GBM samples with high BRAF (*n* = 76) or low BRAF (*n* = 76) levels from the TCGA Glioblastoma Project from cBioPortal. **C** Correlation analysis of METTL3 and BRAF expression in primary and recurrent grade 4 glioma samples from the CGGA database. **D** Correlation analysis of METTL3 and BRAF expression in GBM samples from the TCGA database using GEPIA2. **E** IHC analysis of BRAF V600E, p-ERK, and METTL3 expression in patient-derived GBM samples with or without the BRAF V600E mutation. Scale bar: 400 μm, 100 μm. **F** Western blot analysis of MEK, p-MEK, ERK, p-ERK, and METTL3 protein expression in GSCs with or without BRAF V600E. **G**–**I **GSCs with or without BRAF V600E were treated with CHX (0, 4, 8, and 12 h). Protein expression of ERK, p-ERK, and METTL3 was analyzed by western blot. **J** Co-IP analysis of the interaction between p-ERK and METTL3 in GSCs. Cell lysates were immunoprecipitated with anti-p-ERK and immunoblotted with anti-METTL3. **K **Molecular docking simulation for the METTL3-p-ERK2 complex. **L**–**Q** The results of molecular dynamics simulation, including Rg (L), RMSD (M), RMSF (N), H-bonds (O), coil, β-sheet, turn, and α-helix structures of METTL3 (P), and coil, β-sheet, turn, and α-helix structures of p-ERK (Q). WT, BRAF wild type; V600E, BRAF V600E; TCGA, The Cancer Genome Atlas; CGGA, Chinese Glioma Genome Atlas; CHX, cycloheximide; Rg, radius of gyration; RMSD, root mean square deviation; RMSF, root mean square fluctuation; H-bond, hydrogen bond. *p < 0.05, **p < 0.01, ***p < 0.001

m^6^A modification is one of the most abundant modifications in eukaryotic RNA and is critically involved in cancer initiation and malignant progression [12, 13]. m^6^A dot blot analysis showed a significant increase in dot signal intensity in GSCs (GSC1, GSC2, and GSC0147) and GBM cells (U87MG and U251) expressing BRAF V600E, and quantitative analysis confirmed that m^6^A levels in BRAF V600E-expressing GSCs and GBM cells were significantly higher than those in BRAF wild-type control cells (Fig. 2A, Supplementary Figure S1 A, B).

We investigated the expression of m^6^A modification regulators in GBM patients in the TCGA Glioblastoma Project [34] stratified by BRAF gene expression level. The results revealed significant differences in the expression of various writers, erasers, and readers, between GBM patients with low and high expression of the BRAF gene (Fig. 2B). METTL3 mediates the enrichment of m^6^A methylation modifications [14], while our analysis of the CGGA and TCGA databases revealed a positive correlation between BRAF and METTL3 expression in grade 4 glioma and GBM samples (Fig. 2C,D). However, this analysis lacks stratification based on the patients’ BRAF mutation status. Further, IHC analysis of tumor samples from GBM patients revealed higher protein levels of p-ERK and METTL3 in the BRAF V600E-mutant GBM (Fig. 2E).

Western blot analysis revealed that BRAF V600E led to activation of the ERK signaling pathway, as indicated by increased phosphorylation of its direct substrate MEK and downstream effector ERK, and concurrently increased METTL3 protein levels in GSCs and GBM cells (Fig. 2F, Supplementary Figure S1C, D). Phosphorylated ERK2 (p-ERK2), the major active form of ERK, binds and phosphorylates downstream target proteins, leading to their stabilization [35]. CHX chase assays further showed that BRAF V600E enhanced the stability of METTL3 protein in GSCs and GBM cells (Fig. 2G–I, Supplementary Figure S1E). Co-immunoprecipitation (co-IP) assay indicated that a protein-protein interaction between p-ERK and METTL3 in GSCs and GBM cells (Fig. 2J, Supplementary Figure S1F).

We further investigated whether p-ERK2 directly binds to METTL3. Molecular docking results revealed that p-ERK2 forms a stable complex with METTL3 (Fig. 2K). Molecular dynamics simulations showed that the system’s Rg became stable at 100 ns (Fig. 2L), and RMSD values indicated that the system quickly reached equilibrium and remained stable (Fig. 2M). The RMSF analysis demonstrated that the fluctuation of METTL3’s amino acid residues was greater than that of p-ERK2 (Fig. 2N). Hydrogen bond analysis revealed dynamic interactions between the two proteins, with an average of 10.14 hydrogen bonds (Fig. 2O). Secondary structure analysis showed that the coil, β-sheet, turn, and α-helix structures of both METTL3 and p-ERK2 maintained high stability (Fig. 2P, Q). Therefore, the binding between p-ERK2 and METTL3 rapidly reached equilibrium and maintained a stable conformation. These results demonstrate that BRAF V600E upregulates METTL3 protein level and enhances its stability through ERK activation, thereby facilitating m^6^A methylation enrichment in GSCs and GBM cells. Phosphorylated ERK2 regulates METTL3 protein stability through direct binding.

### BRAF V600E-induced malignant phenotypes of GSCs and GBM cells are METTL3-dependent

**Fig. 3:**
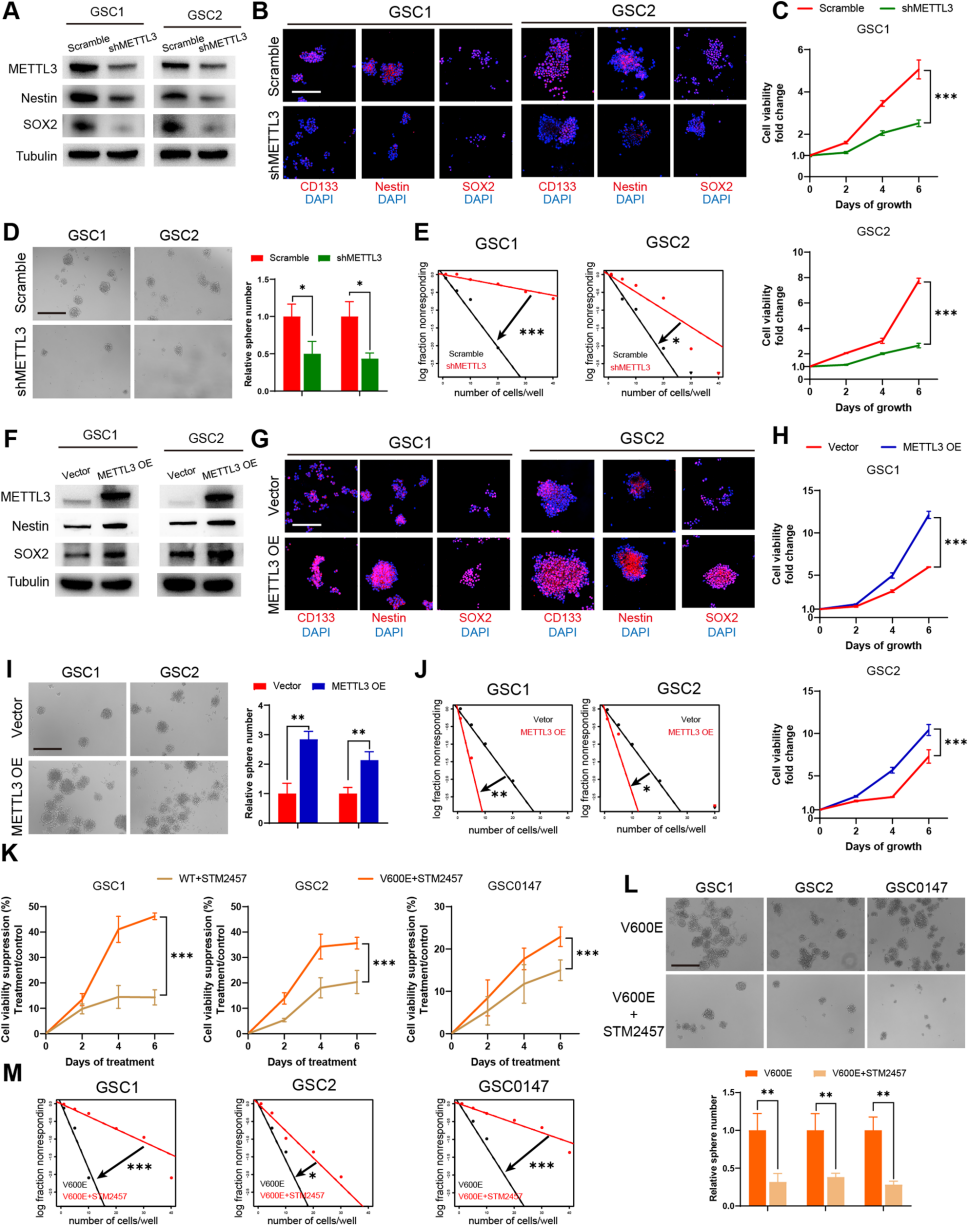
BRAF V600E-induced malignant phenotypes of GSCs and GBM cells are METTL3-dependent. **A** Western blot analysis of Nestin, SOX2 protein expression following METTL3 knockdown in GSCs. **B** Representative immunofluorescence images of Nestin, SOX2, and CD133 in METTL3-knockdown GSCs. Nuclei are labeled with DAPI. Scale bar: 200 μm. **C** Cell viability of METTL3-knockdown GSCs compared with control cells, assessed by CCK-8 assay. **D** Sphere formation of METTL3-knockdown GSCs compared with wild-type controls and quantification of relative sphere numbers. Scale bar: 400 μm. **E** In vitro limiting dilution assay of METTL3-knockdown GSCs compared with wild-type controls. **F **Western blot analysis of Nestin, SOX2 protein expression following METTL3 overexpression in GSCs. **G** Representative immunofluorescence images of Nestin, SOX2, and CD133 in METTL3-overexpressing GSCs. Nuclei are labeled with DAPI. Scale bar: 200 μm. **H** Cell viability of METTL3-overexpressing GSCs compared with control cells, assessed by CCK-8 assay. **I** Sphere formation of METTL3-overexpressing GSCs compared with wild-type controls and quantification of relative sphere numbers. Scale bar: 400 μm. **J** In vitro limiting dilution assay of METTL3-overexpressing GSCs compared with wild-type controls. **K** Cell viability suppression assays illustrating the sensitivity of GSCs with or without BRAF V600E to STM2457, assessed by CCK-8 assay. **L** Sphere formation assays illustrating the sensitivity of BRAF V600E-expressing GSCs to STM2457 and quantification of relative sphere numbers. Scale bar: 400 μm. **M** In vitro limiting dilution assays illustrating the sensitivity of BRAF V600E-expressing GSCs to STM2457. WT, BRAF wild type; V600E, BRAF V600E. *p < 0.05, **p < 0.01, ***p < 0.001

To determine whether METTL3 is involved in the stemness of GSCs, we constructed METTL3-knockdown GSCs using shRNA lentiviral transfection. Efficient knockdown was confirmed by western blot analysis (Fig. 3A). Knockdown of METTL3 markedly reduced the expression of Nestin and SOX2 in GSCs (Fig. 3A). Immunofluorescence staining revealed decreased fluorescence intensities of CD133, Nestin, and SOX2 in METTL3-knockdown GSCs (Fig. 3B). METTL3 knockdown significantly impaired GSC proliferation (Fig. 3C), diminished tumor sphere formation (Fig. 3D), and reduced self-renewal capacity as assessed by limiting dilution assays (Fig. 3E). In contrast, METTL3 overexpression in GSCs using lentiviral transfection, validated by western blotting (Fig. 3F), increased the protein levels of Nestin and SOX2 (Fig. 3F) and enhanced the fluorescence intensities of CD133, Nestin, and SOX2 (Fig. 3G). METTL3 overexpression significantly promoted GSCs proliferation (Fig. 3H), increased sphere-forming efficiency (Fig. 3I), and augmented self-renewal ability (Fig. 3J).

We next examined whether METTL3 is involved in the invasiveness of GBM cells by generating METTL3-knockdown U87MG and U251 cells (Supplementary Figure S2A). METTL3 knockdown significantly reduced the proliferation and migratory ability of GBM cells (Supplementary Figure S2B, C). We overexpressed METTL3 in U87MG and U251 cells (Supplementary Figure S2D). METTL3 overexpression significantly promoted the proliferation and migration ability of GBM cells (Supplementary Figure S2E, F). These results indicate that METTL3 promotes the stemness of GSCs and the invasiveness of GBM cells in vitro.

To investigate whether the regulation of malignant phenotypes in BRAF V600E-expressing GBM cells is dependent on the methyltransferase activity of METTL3, we treated GBM cells with the specific METTL3 inhibitor STM2457 targeting methyltransferase activity [36]. We first determined the IC50 of STM2457 in GBM cells (Supplementary Figure S2G). To assess the treatment sensitivity, we performed subsequent experiments at sub-IC50 concentrations of STM2457. We observed that the inhibitory effect of STM2457 was significantly greater in BRAF V600E-expressing cells than in BRAF wild-type control cells (Fig. 3K, Supplementary Figure S2H). These data indicate that BRAF V600E-expressing GSCs and GBM cells are more sensitive to STM2457. STM2457 also strongly suppressed the sphere-forming capability (Fig. 3L) and self-renewal potential (Fig. 3M) of BRAF V600E-expressing GSCs. STM2457 exerted a more pronounced inhibitory effect on the migration of BRAF V600E-expressing GBM cells (Supplementary Figure S2I). These results suggest that the effect of BRAF V600E on the stemness of GSCs and the invasiveness of GBM cells is dependent on the methyltransferase activity of METTL3.

### METTL3 stabilizes BRAF mRNA in an m^6^A-dependent manner to Establish a positive feedback loop

**Fig. 4:**
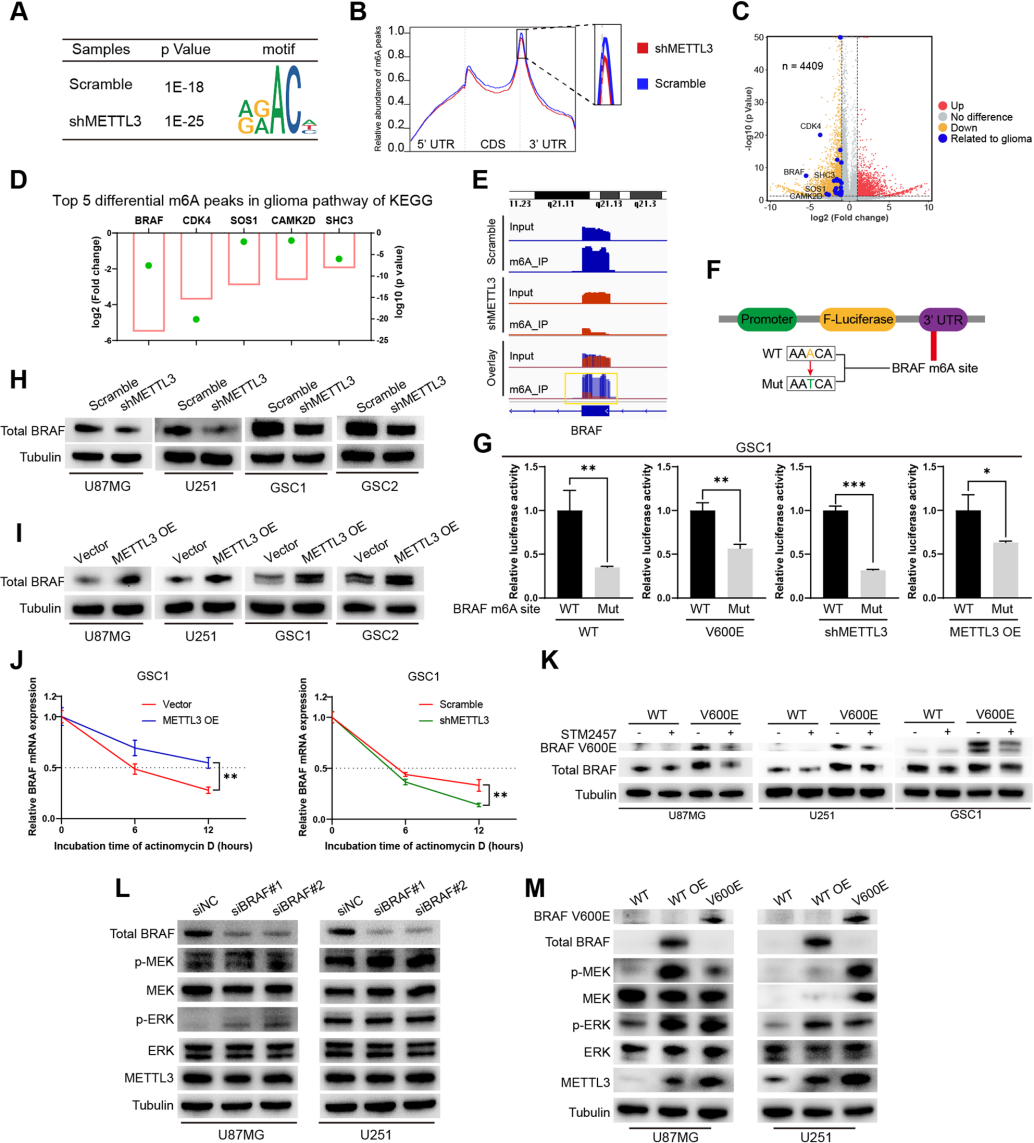
METTL3 stabilizes BRAF mRNA in an m^6^A-dependent manner to establish a positive feedback loop. **A** The consensus RRACH motif was identified in GBM cells with or without METTL3 knockdown, as determined by MeRIP-seq. **B** Distribution of m^6^A peaks across mRNA transcripts, with relative abundance of m^6^A peaks determined. **C **Volcano plot showing differential m^6^A peaks between METTL3 knockdown and control cells. **D **Top five genes with significantly downregulated m^6^A peaks in glioma pathways of KEGG, identified by MeRIP-seq. The green point shows the log10 (*p* value), and the red box shows the log2 (fold change). **E** m^6^A peak visualization of BRAF mRNA with or without METTL3 knockdown. **F** Schematic representation of the wild-type or mutant m^6^A BRAF constructs in the firefly luciferase reporter vector. **G** Relative luciferase activity of wild-type and mutant BRAF 3′ UTR reporter vectors in GSCs under different genetic backgrounds: with or without BRAF V600E expression, and with METTL3 knockdown or overexpression. **H** Total BRAF protein levels in GBM cells and GSCs with or without METTL3 knockdown, as analyzed by western blot. **I** Total BRAF protein levels in GBM cells and GSCs with or without METTL3 overexpression, as analyzed by western blot. **J** Stability of BRAF mRNA in GSCs with METTL3 overexpression or knockdown, following transcriptional inhibition by actinomycin D. **K** Total BRAF and BRAF V600E protein expression in GBM cells and GSCs after treatment with STM2457, as analyzed by western blot.  WT, BRAF wild type; V600E, BRAF V600E. *p < 0.05, **p < 0.01, ***p < 0.001

To identify the genes whose m^6^A levels are regulated by METTL3, we performed MeRIP-seq on U87MG cells with or without METTL3 knockdown. The results showed that the m^6^A peaks in both METTL3 knockdown and control samples shared the same motif, which matched the consensus sequence (RRACH) characteristic of m^6^A modification (Fig. 4A). The distribution pattern showed that m^6^A peaks were particularly enriched in the 3′ UTR near the mRNA stop codon. After METTL3 knockdown, the abundance of m^6^A peaks decreased at various positions along the mRNA (Fig. 4B), reflecting the regulatory role of METTL3 expression on m^6^A modification. We focused on the m^6^A peaks whose levels were downregulated following METTL3 knockdown. A volcano plot displaying the differential m^6^A peaks between samples with or without METTL3 knockdown is shown in Fig. 4C. We identified 4409 m^6^A peaks whose methylation levels were significantly downregulated after METTL3 knockdown, and several differential peaks were associated with glioma (Fig. 4C), suggesting that METTL3 may regulate glioma progression via m^6^A modification. The top five differentially downregulated m^6^A peaks in the glioma pathway were associated with the genes BRAF, CDK4, SOS1, CAMK2D, and SHC3, with the m^6^A peak of BRAF showing the most significant downregulation (Fig. 4D). Visualization of the m^6^A peaks revealed that BRAF mRNA was enriched with m^6^A peaks, and these peaks were significantly downregulated following METTL3 knockdown (Fig. 4E).

To investigate whether m^6^A modification affects BRAF expression, we cloned wild-type and mutant m^6^A sites of BRAF mRNA into the 3′ UTR downstream of the firefly luciferase coding region in a reporter vector (Fig. 4F). Luciferase results in GSCs and GBM cells showed that compared with the reporter vector harboring the wild-type m^6^A site, which is susceptible to methylation, the mutant m^6^A site significantly inhibited luciferase activity regardless of the BRAF mutation status and METTL3 expression levels in cells (Fig. 4G, Supplementary Figure S3A–C). These findings suggested that m^6^A modification on BRAF mRNA possess the function of regulating BRAF expression. Western blot analysis showed that knockdown of METTL3 in U87MG, U251, GSC1, and GSC2 cells inhibited BRAF protein expression (Fig. 4H), while overexpression of METTL3 promoted BRAF protein expression (Fig. 4I). While METTL3 knockdown promoted BRAF mRNA decay, overexpression of METTL3 increased the stability of BRAF mRNA (Fig. 4J, Supplementary Figure S3D). Our findings indicate that METTL3 stabilizes BRAF mRNA in GSCs and GBM cells through m^6^A modification, thereby increasing BRAF expression.

In cells inhibited for METTL3 activity by STM2457, the total BRAF protein and BRAF V600E protein levels were reduced, with this change being more pronounced in BRAF V600E-expressing cells (Fig. 4K). These results demonstrate that the expression of BRAF V600E is more dependent on METTL3. Together these findings suggest that METTL3 has a positive feedback effect on the expression of BRAF V600E. The positive feedback loop between BRAF V600E and METTL3 maintains METTL3 expression, thereby driving the stemness of GSCs and the invasiveness of GBM cells.

To ascertain the role of wild-type BRAF in regulating the MEK/ERK pathway and METTL3 expression in GBM, we performed both knockdown and overexpression experiments. While wild-type BRAF knockdown significantly inhibited the viability and increased the proportion of dead cells of GBM cells, it did not significantly alter the basal activity of the MEK/ERK pathway and the expression of METTL3 (Supplementary Figure S4A–C). Wild-type BRAF overexpression activated the pathway, as evidenced by increased phosphorylation of MEK and ERK. This activation led to the upregulation of METTL3 (Supplementary Figure S4D). These data indicate that whether wild-type BRAF forms a positive feedback loop with METTL3 depends on whether its expression level is sufficiently high to activate the ERK signaling pathway.

### METTL3 regulates the m^6^A modification of SQSTM1 and induces autophagy

**Fig. 5:**
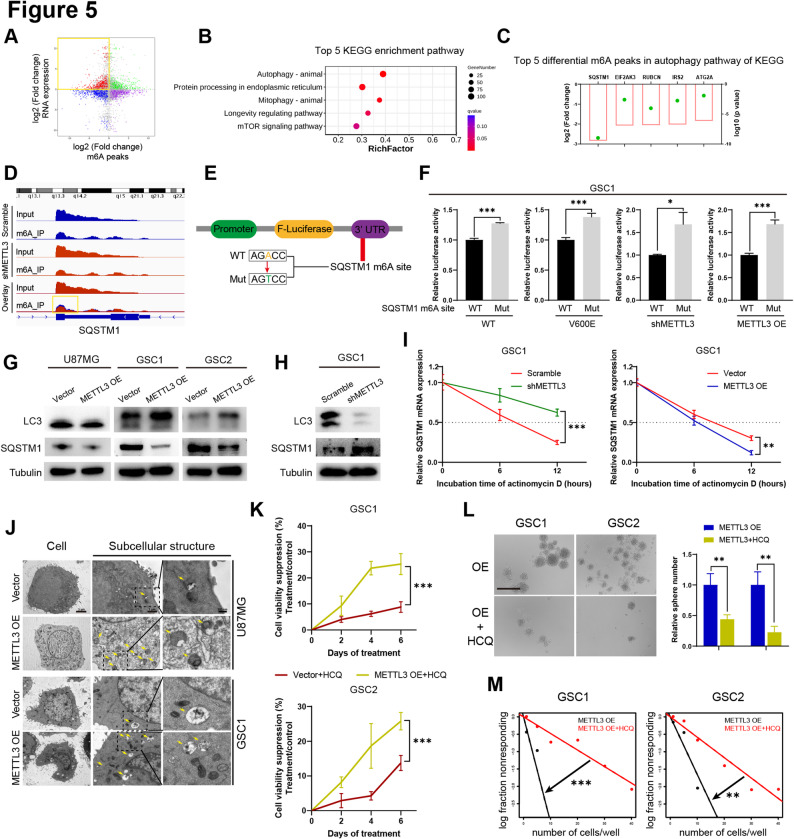
METTL3 regulates the m^6^A modification of SQSTM1 and induces autophagy. **A** Four-quadrant plot of RNA expression and m^6^A peak co-analysis. **B** KEGG enrichment analysis in accordance with the red dots in the yellow box in (A). **C **Top five genes with significantly downregulated m^6^A peaks in autophagy pathways of KEGG, identified by MeRIP-seq. The green point shows the log10 (*p* value), and the red box shows the log2 (fold change). **D** m^6^A peak visualization of SQSTM1 mRNA with or without METTL3 knockdown. **E** Schematic representation of the wild-type or mutant m^6^A SQSTM1 constructs in the firefly luciferase reporter vector. **F** Relative luciferase activity of wild-type and mutant SQSTM1 3′ UTR reporter vectors in GSCs under different genetic backgrounds: with or without BRAF V600E expression, and with METTL3 knockdown or overexpression. **G** LC3 and SQSTM1 protein levels in GBM cells and GSCs with or without METTL3 overexpression, as analyzed by western blot. **H** Stability of SQSTM1 mRNA in GSCs with METTL3 overexpression or knockdown, following transcriptional inhibition by actinomycin D. **I** LC3 and SQSTM1 protein levels in GSCs with or without METTL3 knockdown, as analyzed by western blot.  **J** Representative TEM images showing autophagosomes in GBM cells and GSCs with or without METTL3 overexpression. Yellow arrow: autophagosome. Scale bars: 3 μm, 1 μm, 300 nm. **K** Cell viability suppression assays illustrating the sensitivity of GSCs with or without METTL3 overexpression to HCQ, assessed by CCK-8 assay. **L **Sphere formation assays illustrating the sensitivity of GSCs with METTL3 overexpression to HCQ and quantification of relative sphere numbers. Scale bar: 400 μm. **M** In vitro limiting dilution assay illustrating the sensitivity of GSCs with METTL3 overexpression to HCQ. WT, BRAF wild type; V600E, BRAF V600E; HCQ, hydroxychloroquine. *p < 0.05, **p < 0.01, ***p < 0.001

The MeRIP-seq and RNA-seq data revealed that the expression of certain genes associated with downregulated m^6^A peaks was increased (red dots within the yellow box, Fig. 5A), suggesting that methylation modification of these genes potentially exerted a negative regulatory effect on RNA expression levels. KEGG enrichment analysis of these genes revealed that autophagy was the most significantly associated pathway (Fig. 5B). In the autophagy pathway, the top five genes with the most significant decrease in m^6^A levels were SQSTM1, EIF2AK3, RUBCN, IRS2, and ATG2A (Fig. 5C). Visualization of the m^6^A peak in SQSTM1 showed that METTL3 knockdown significantly downregulated the m^6^A peak of SQSTM1 (Fig. 5D), indicating that the presence of highly active m^6^A sites on SQSTM1 mRNA.

To determine whether m^6^A modification of SQSTM1 mRNA regulates its expression, we performed luciferase assays using wild-type and m^6^A -mutated sequences (Fig. 5E). Mutation of the SQSTM1 m^6^A sites resulted in increased luciferase activity (Fig. 5F, Supplementary Figure S5A–C.), suggesting that m^6^A modification on SQSTM1 mRNA negatively regulated SQSTM1 expression. METTL3 overexpression decreased SQSTM1 protein levels through accelerating mRNA decay (Fig. 5G, H). Knockdown of METTL3 increased SQSTM1 protein (Fig. 5I) through inhibiting the SQSTM1 mRNA decay (Fig. 5H, Supplementary Figure S5D). These findings suggest that METTL3 negatively regulates SQSTM1 expression in GSCs and GBM cells through m^6^A modification.

SQSTM1 is a key regulator of autophagy [37]. Western blot confirmed that METTL3 overexpression promotes autophagy in GSCs, as evidenced by increased LC3 protein levels (Fig. 5G). Knockdown of METTL3 in GSCs resulted in decreased LC3 levels (Fig. 5I). TEM images revealed that the number of autophagosomes was markedly increased in METTL3-overexpressing GSCs and GBM cells (Fig. 5J). To determine whether METTL3 promotes the stemness of GSCs through autophagy activation, we treated GSCs with the autophagy inhibitor HCQ. The inhibitory effect of HCQ was significantly greater in METTL3-overexpressing cells than in control cells (Fig. 5K). In METTL3-overexpressing GSCs, treatment with HCQ resulted in attenuated sphere-forming capacity and self-renewal potential (Fig. 5L, M). These results suggest that METTL3-mediated m^6^A modification activates autophagy by inhibiting SQSTM1 expression, consequently enhancing GSCs’ stemness.

**Fig. 6:**
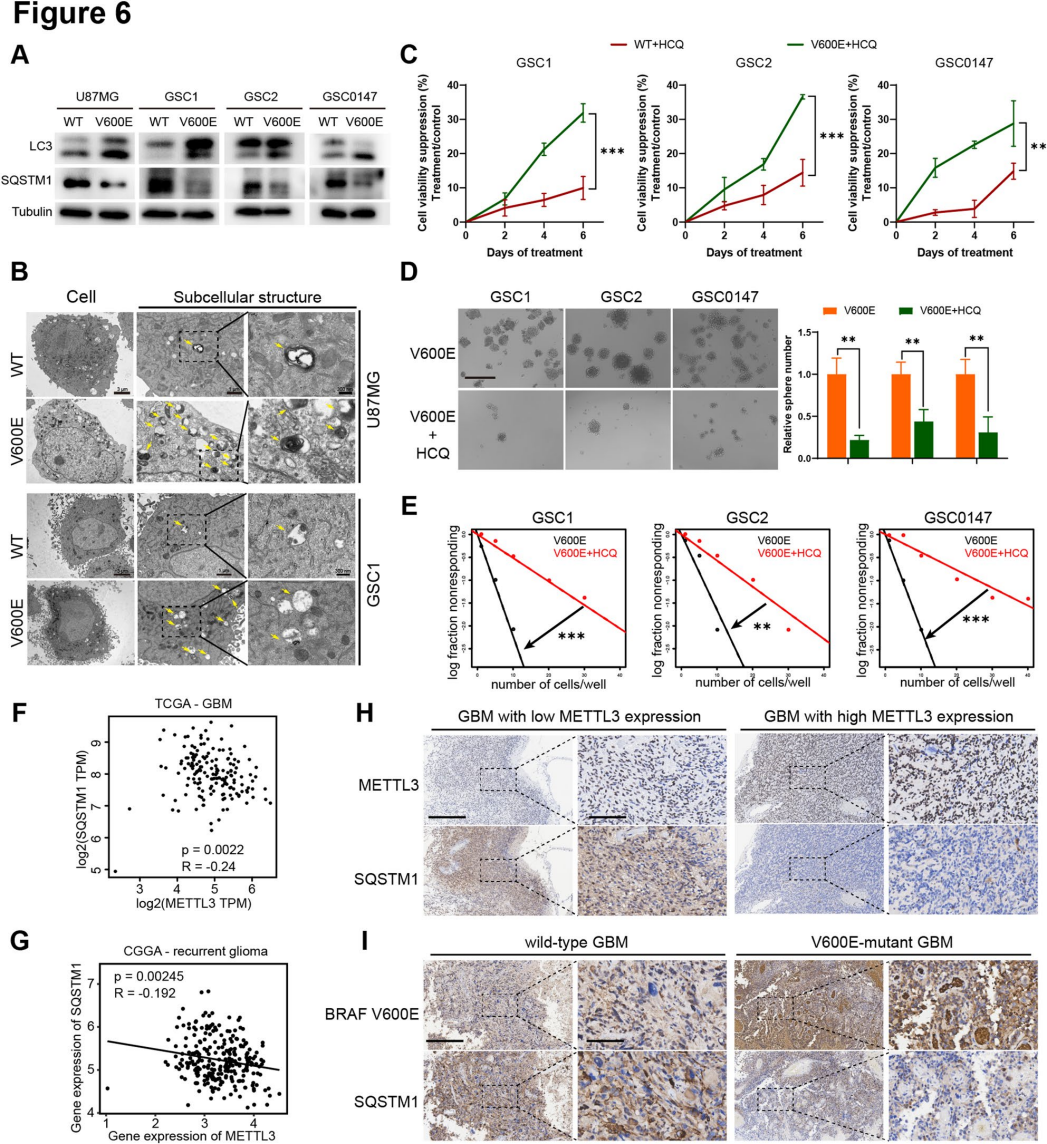
BRAF V600E-driving stemness of GSCs is autophagy-dependent. **A** LC3 and SQSTM1 protein levels in GBM cells and GSCs with or without BRAF V600E, as analyzed by western blot. **B** Representative TEM images showing autophagosomes in GBM cells and GSCs with or without BRAF V600E. Yellow arrow: autophagosome. Scale bars: 3 μm, 1 μm, 300 nm. **C** Cell viability suppression assays illustrating the sensitivity of GSCs with or without BRAF V600E to HCQ, assessed by CCK-8 assay. **D** Sphere formation assays illustrating the sensitivity of BRAF V600E-expressing GSCs to HCQ and quantification of relative sphere numbers. Scale bar: 400 μm. **E** In vitro limiting dilution assay illustrating the sensitivity of BRAF V600E-expressing GSCs to HCQ. **F** Correlation analysis of METTL3 and SQSTM1 expression in GBM samples from the TCGA database using GEPIA2. **G** Correlation analysis of METTL3 and SQSTM1 expression in recurrent glioma samples from the CGGA database. **H** IHC analysis of METTL3 and SQSTM1 expression in patient-derived GBM samples with high or low METTL3 expression. **I** IHC analysis of BRAF V600E and SQSTM1 expression in patient-derived GBM samples with or without the BRAF V600E mutation. Scale bar: 400 μm, 100 μm. WT, BRAF wild type; V600E, BRAF V600E; HCQ, hydroxychloroquine. *p < 0.05, **p < 0.01, ***p < 0.001

We examined the role of the BRAF V600E-METTL3 axis in regulating autophagy. Western blot analysis confirmed that BRAF V600E promotes autophagy in GSCs and GBM cells, as evidenced by decreased SQSTM1 and increased LC3 protein levels (Fig. 6A). TEM images revealed that the number of autophagosomes in BRAF V600E-expressing GSCs and GBM cells was significantly higher than in control cells (Fig. 6B). To examine whether BRAF V600E promotes the stemness of GSCs and the invasiveness of GBM cells through autophagy activation, we treated cells with HCQ. BRAF V600E-expressing cells exhibited a significantly increased sensitivity to HCQ-induced apoptosis and proliferation inhibition (Fig. 6C, Supplementary Figure S5E, G). Additionally, their sphere-forming, self-renewal, and migratory ability are autophagy-dependent (Fig. 6D, E, Supplementary Figure S5F).

Database analyses revealed a negative correlation between METTL3 and SQSTM1 expression in the TCGA cohort (Fig. 6F) and in the CGGA cohort (Fig. 6G). Consistent with our mechanistic findings, IHC analysis of GBM samples revealed an inverse correlation between METTL3 and SQSTM1 protein levels (Fig. 6H). This relationship was also observed in the BRAF V600E mutant GBM samples, which exhibited significantly reduced SQSTM1 expression (Fig. 6I). Taken together, our findings suggest that BRAF V600E-driven METTL3 expression activates autophagy by inhibiting SQSTM1 expression. This pathway plays a crucial role in the stemness of GSCs.

### Inhibition of METTL3 or autophagy reverses BRAF V600E-mediated GBM progression in vivo

**Fig. 7:**
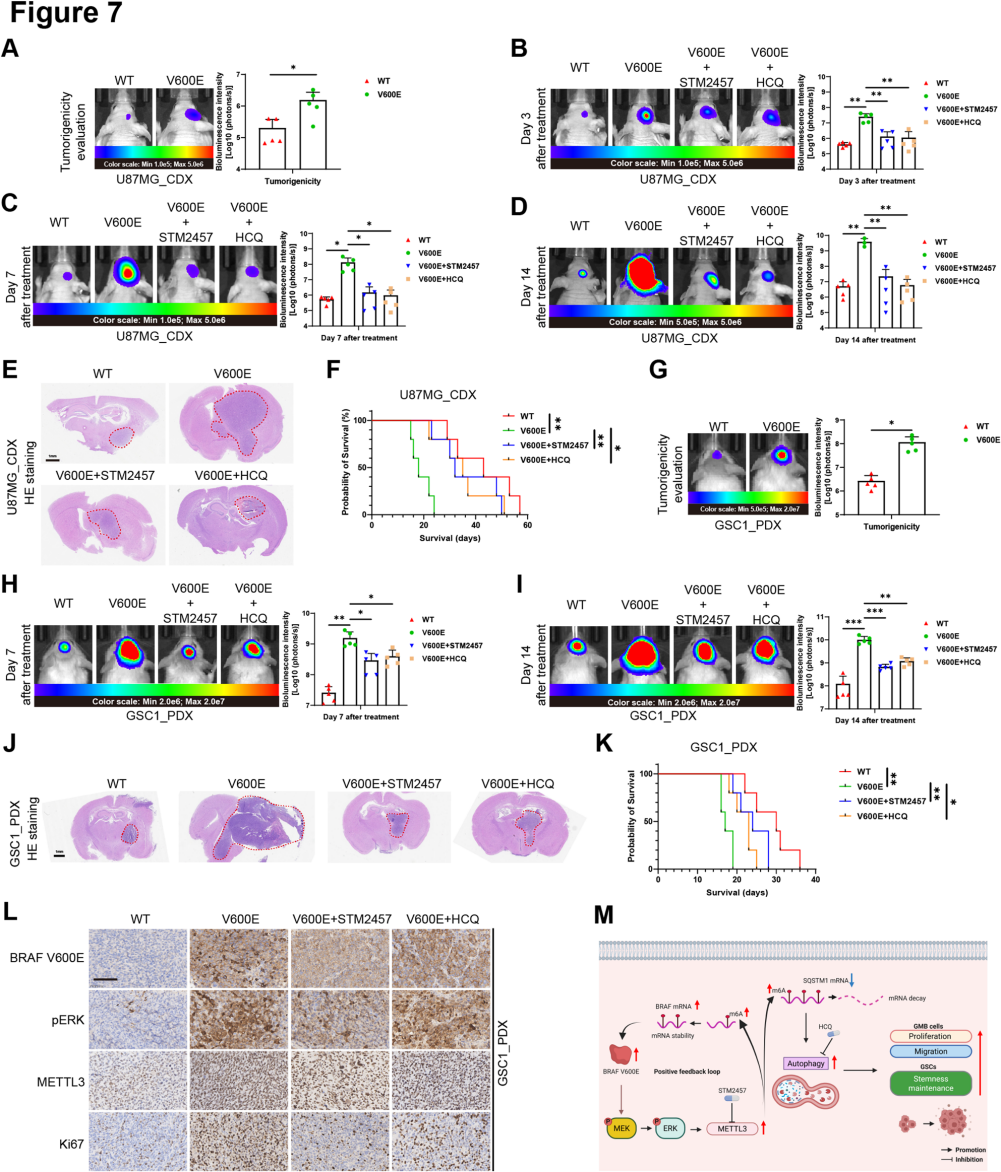
Inhibition of METTL3 or autophagy reverses BRAF V600E-mediated GBM progression in vivo. **A** Tumorigenicity assessment: representative IVIS images and quantification of bioluminescence intensity of tumors in orthotopic CDX models (5 mice per group). **B**–**D **Representative IVIS images and bioluminescence intensity of tumors in orthotopic CDX models on day 3, day 7, and day 14 after treatment with STM2457 or HCQ (5 mice per group). **E** Representative HE staining images of brains from tumor-bearing mouse CDX models. Scale bars: 1 mm. **F** Survival curves of orthotopic mouse CDX models in the indicated groups (5 mice per group). **G** Tumorigenicity assessment: representative IVIS images and quantification of bioluminescence intensity of tumors in orthotopic PDX models (5 mice per group). **H**–**I** Representative IVIS images and bioluminescence intensity of tumors in orthotopic PDX models on day 7 and day 14 after treatment with STM2457 or HCQ (5 mice per group). **J** Representative HE staining images of brains from tumor-bearing mouse PDX models. Scale bars: 1 mm. **K** Survival curves of orthotopic mouse PDX models in the indicated groups (5 mice per group). **L **Representative IHC staining images of BRAF V600E, p-ERK, METTL3 and Ki67 expression in tumors from tumor-bearing mouse PDX models, Scale bars: 100 μm. **M **Schematic diagram of the mechanism of this study. Created in BioRender.com. WT, BRAF wild type; V600E, BRAF V600E; HCQ, Hydroxychloroquine; CDX, cell-derived xenografts; PDX, patient-derived xenografts. *p < 0.05, **p < 0.01, ***p < 0.001

To investigate whether the BRAF V600E drives tumor progression of GBM in vivo, we constructed the mouse orthotopic CDX models by intracranial injection of U87MG cells. In vivo imaging revealed that the tumorigenicity of BRAF V600E-expressing GBM cells was significantly stronger than that of BRAF wild-type control cells (Fig. 7A). We next explored whether inhibiting METTL3 function or blocking autophagy could prevent tumor progression in vivo driven by BRAF V600E. Bioluminescence imaging of tumors demonstrated that the volume of BRAF V600E-expressing GBM tumors was significantly reduced in both the STM2457 treatment group and the HCQ treatment group at day 3, day 7, and day 14 post-treatment (Fig. 7B–D). Analysis of brain sections revealed that the tumor volume in the BRAF V600E-expressing group was larger than that in the BRAF wild-type control group, while the tumor volume decreased after STM2457 or HCQ treatment (Fig. 7E). Furthermore, while BRAF V600E significantly shortened the survival of tumor-bearing mice, STM2457 or HCQ effectively reversed the effects of BRAF V600E on mouse survival (Fig. 7F).

To validate the generality of these findings, we replicated the in vivo experiments in the mouse orthotopic PDX models by using patient-derived GSC1 cells. BRAF V600E expression in GSCs robustly enhanced tumorigenicity (Fig. 7G). The therapeutic efficacy of STM2457 and HCQ was also confirmed, as both inhibitors significantly suppressed tumor growth at day 7 and day 14 post-treatment (Fig. 7H, I), reduced tumor burden (Fig. 7J), and conferred a significant survival advantage (Fig. 7K). IHC staining indicated that tumor with expression of BRAF V600E also exhibited high expression of p-ERK and METTL3. Moreover, Ki67 staining intensity and the number of positive cells was higher in the BRAF V600E tumors, suggesting that BRAF V600E enhanced the proliferative capacity of GBM in vivo, while STM2457 or HCQ treatment reduced the number of Ki67-positive cells (Fig. 7L, Supplementary Figure S6A). These in vivo results indicate that BRAF V600E drives malignant progression of orthotopic GBM in mice, shortening survival, while inhibition of METTL3 or autophagy reverses the detrimental effects of BRAF V600E.

## Discussion

GBM is the most common primary malignant brain tumor in adults, with invasive growth in the brain, leading to a short survival time [1, 2, 38]. The molecular heterogeneity of GBM poses challenges for precision therapy [3–5], highlighting the need to identify key molecular factors driving GBM progression. The BRAF V600E mutation is associated with the tumorigenesis and progression of various cancers [39–42], but its impact on GBM remains unclear. Approximately 70% of eGBMs harbor the BRAF V600E mutation [9], and eGBM patients show significantly worse prognosis compared with patients with other GBM subtypes [10]. This suggests that BRAF V600E may promote GBM progression, warranting further research for validation. GSCs represent a critical cellular subset that initiates GBM progression and recurrence [43]. In this study, we confirmed that BRAF V600E enhances the expression of stemness markers, proliferation, migration, sphere formation, and self-renewal in GSCs or GBM cells.

Elevated BRAF kinase activity serves as a marker for GBM progression and recurrence [11]. BRAF V600E is the most active isoform of BRAF kinase and constitutively activates the RAF/MEK/ERK signaling pathway [7, 8], but the detailed mechanism through which it regulates GBM progression remains largely unknown. Numerous studies have demonstrated that m^6^A modification and the dysregulation of m^6^A-related factors (writers, erasers, and readers) are implicated in various types of cancer and mediate cancer development, metastasis, drug resistance, and immune evasion [14, 44]. METTL3 is one of the most important m^6^A methyltransferases [14]. Our analysis of TCGA and CGGA databases revealed a positive correlation between BRAF and METTL3 expression in GBM patients, and we observed high METTL3 expression levels in BRAF V600E-mutated GBM samples from patients. A previous report showed that active ERK proteins can phosphorylate METTL3, thereby inhibiting its ubiquitin-dependent proteasomal degradation [35]. Our findings indicate that BRAF V600E in GSCs and GBM cells, through ERK signaling activation, promotes both the expression and stability of METTL3 protein, therefore inducing the enrichment of m^6^A modification.

METTL3 participates in the regulation of cell proliferation, stemness maintenance, and drug resistance through its impact on the fate of target RNAs, although findings for GBM have been contradictory [17, 18, 45, 46]. This could be because of the complex, multifactorial regulation of METTL3 function and the high heterogeneity of GBM. To determine the functional role of METTL3 in GSCs and GBM cells, we modulated METTL3 expression. The results demonstrate that METTL3 plays a key role in promoting malignant biological behaviors such as cell proliferation, migration, sphere-forming capacity, and stemness maintenance. Furthermore, using specific inhibitors targeting the methyltransferase activity of METTL3, we found that the regulatory effect of BRAF V600E on GBM invasiveness and stemness depends on the m^6^A modification. MeRIP-seq found that the m^6^A modification of BRAF mRNA is regulated by METTL3, and we confirmed that METTL3 promotes the expression of total BRAF, particularly the BRAF V600E mutant. This discovery reveals the existence of a positive feedback loop between BRAF V600E and METTL3 in GBM. Previous studies have indicated that positive feedback loops are critical in the malignant progression of cancers [47, 48]. Understanding the mechanisms that sustain oncogenic BRAF V600E signaling in GSCs and GBM cells provides new insights for developing targeted therapies against BRAF V600E-mutated GBM.

Our results showed that when the expression of wild-type BRAF kinase was high enough to induce ERK signaling pathway activation, corresponding changes in METTL3 expression were observed. This finding implied that such a positive feedback loop may function in BRAF wild-type GBM exhibiting high BRAF expression. Although knockdown of wild-type BRAF affected the proliferation and survival of GBM cells, no impact on MEK/ERK signaling pathway activity or METTL3 expression was observed, indicating that ERK activation and METTL3 expression are not dependent on wild-type BRAF. Furthermore, since the activation of wild-type BRAF depends on upstream signaling stimuli [49], in its non-activated state, it may not be the limiting factor for maintaining basal ERK activity. Therefore, the establishment of a feedback loop with METTL3 and wild-type BRAF may require specific conditions.

The relationship between autophagy and the progression, metastasis, and drug resistance has been demonstrated in various cancers [50, 51]. Particularly, autophagy has been demonstrated to promote stemness and therapy resistance in GSCs [26, 27]. Although some research suggests that autophagy inhibits tumor growth [52], central nervous system tumors with the BRAF V600E mutation rely on autophagy activation [28, 53]. However, the regulatory network connecting BRAF V600E and autophagy remains unelucidated. Our results showed that METTL3 downregulates SQSTM1 expression through m^6^A modification, thereby inducing autophagy. Furthermore, GSCs with METTL3 overexpression showed heightened sensitivity to the autophagy inhibitor. BRAF V600E-expressing GSCs exhibited decreased SQSTM1 expression, enhanced autophagy, and significant responsiveness to autophagy inhibitor treatment. Evidence from external databases and GBM samples demonstrated a negative correlation between METTL3 and SQSTM1 expression, with BRAF V600E-mutated GBM exhibiting lower SQSTM1 levels compared with wild-type GBM, which aligns with our experimental findings. SQSTM1, also known as p62, is a classical autophagy receptor essential for the autophagic process [54], and its levels are usually negatively correlated with autophagic flux [55]. SQSTM1 plays a critical role in mTORC1 activation, and mTORC1 inactivation enhances autophagy [37, 56]. Therefore, inhibiting high levels of SQSTM1 favors the increase in autophagic flux by regulating mTORC1. This study reveals that BRAF V600E regulates the proliferation and self-renewal of GSCs through the METTL3-SQSTM1-autophagy axis, thereby mediating GBM progression.

In addition to evidence from GSCs and GBM samples, our results from mouse orthotopic CDX and PDX models demonstrated that both the METTL3 inhibitor and the autophagy inhibitor exerted antitumor effects on BRAF V600E-expressing GBM. However, given the relatively rare occurrence of the BRAF V600E mutation in GBM patients, we were unable to perform further analyses in large-scale GBM cohorts and corresponding clinical data. Our results are primarily based on in vitro experiments, and while therapeutic efficacy was observed in orthotopic PDX models, the development of novel therapeutic targets requires validation through rigorous clinical trials. Additionally, although high-grade gliomas harboring the BRAF V600E mutation show a therapeutic response to BRAF inhibitors [57–59], clinical challenges such as acquired resistance and toxicity remain significant concerns [9, 60–62]. The implications of our findings for BRAF inhibitor resistance research warrant further in-depth investigation.

This study reveals a positive feedback loop between BRAF V600E and METTL3 that mediates autophagy activation, enhanced invasiveness in GBM cells, and reinforced stemness in GSCs, thereby promoting GBM progression.

## Conclusion

In this study, we identified the BRAF V600E/MEK/ERK/METTL3 positive feedback loop. that regulates SQSTM1 to activate autophagy in an m^6^A-dependent manner, ultimately promoting invasiveness and stemness in GBM (Fig. 7M). These findings suggest that METTL3 and autophagy represent potential therapeutic targets for BRAF V600E-mutated GBM, providing crucial preclinical evidence for novel treatment strategies.

## Supplementary Information

Supplementary Material 1.

## Data Availability

The data generated in this study are found in the present article or in supplementary sections. Any additional information is available from the corresponding authors on reasonable request.

## Abbreviations

GBM: glioblastoma
eGBM: epithelioid glioblastoma
m^6^A: N6-methyladenosine
GSCs: glioma stem-like cells
HCQ: Hydroxychloroquine
CHX: Cycloheximide
qPCR: quantitative real-time PCR
IC50: Half-maximal inhibitory concentration
Rg: Radius of gyration
RMSD: Root mean square deviation
RMSF: Root mean square fluctuation
H-bond: Hydrogen bond
MeRIP-seq: Methylated RNA immunoprecipitation sequencing
RNA-seq: RNA sequencing
3′ UTR, 3: Untranslated region
KEGG: Kyoto encyclopedia of genes and genomes
TEM: Transmission electron microscopy
IVIS: In vivo imaging system
TCGA: The Cancer Genome Atlas
CGGA: Chinese Glioma Genome Atlas
CDX: Cell-derived xenografts
PDX: Patient-derived xenografts

## References

[1] Ostrom QT, et al. CBTRUS statistical report: primary brain and other central nervous system tumors diagnosed in the united States in 2016–2020. Neuro Oncol. 2023;25(12 Suppl 2):iv1–99. 37793125 10.1093/neuonc/noad149 PMC10550277

[2] Wen PY, et al. Glioblastoma in adults: a society for Neuro-Oncology (SNO) and European society of Neuro-Oncology (EANO) consensus review on current management and future directions. Neurooncol. 2020;22(8):1073–113. 10.1093/neuonc/noaa106 PMC7594557 32328653

[3] Mathur R, et al. Glioblastoma evolution and heterogeneity from a 3D whole-tumor perspective. Cell. 2024;187(2):446–e46316. 38242087 10.1016/j.cell.2023.12.013 PMC10832360

[4] Dakal TC, Kakde GS, Maurya PK. Genomic, epigenomic and transcriptomic landscape of glioblastoma. Metab Brain Dis. 2024;39(8):1591–611. 39180605 10.1007/s11011-024-01414-8

[5] Le Rhun E, et al. Molecular targeted therapy of glioblastoma. Cancer Treat Rev. 2019;80:101896. 31541850 10.1016/j.ctrv.2019.101896

[6] Dankner M, et al. Classifying BRAF alterations in cancer: new rational therapeutic strategies for actionable mutations. Oncogene. 2018;37(24):3183–99. 29540830 10.1038/s41388-018-0171-x

[7] Davies H, et al. Mutations of the BRAF gene in human cancer. Nature. 2002;417(6892):949–54. 12068308 10.1038/nature00766

[8] Pratilas CA, et al. (V600E)BRAF is associated with disabled feedback Inhibition of RAF-MEK signaling and elevated transcriptional output of the pathway. Proc Natl Acad Sci U S A. 2009;106(11):4519–24. 19251651 10.1073/pnas.0900780106 PMC2649208

[9] Andrews LJ, et al. Prevalence of BRAFV600 in glioma and use of BRAF inhibitors in patients with BRAFV600 mutation-positive glioma: systematic review. Neuro Oncol. 2022;24(4):528–40. 34718782 10.1093/neuonc/noab247 PMC8972326

[10] Xi S, et al. Adult epithelioid glioblastoma exhibits an extremely poor prognosis and high frequency of SWI/SNF complex mutation: insights from a retrospective study. Int J Cancer. 2024;155(1):172–83. 38411299 10.1002/ijc.34854

[11] Kim KH, et al. Integrated proteogenomic characterization of glioblastoma evolution. Cancer Cell. 2024;42(3):358–e3778. 38215747 10.1016/j.ccell.2023.12.015 PMC10939876

[12] An Y, Duan H. The role of m<Superscript>6</Superscript>a RNA methylation in cancer metabolism. Mol Cancer. 2022;21(1):14. 35022030 10.1186/s12943-022-01500-4 PMC8753874

[13] Boulias K, Greer EL. Biological roles of adenine methylation in RNA. Nat Rev Genet. 2023;24(3):143–60. 36261710 10.1038/s41576-022-00534-0 PMC9974562

[14] Deng X, et al. The roles and implications of RNA m(6)A modification in cancer. Nat Rev Clin Oncol. 2023;20(8):507–26. 37221357 10.1038/s41571-023-00774-x PMC12466201

[15] Wang Y, et al. N6-methyladenosine modification destabilizes developmental regulators in embryonic stem cells. Nat Cell Biol. 2014;16(2):191–8. 24394384 10.1038/ncb2902 PMC4640932

[16] Jia G, et al. N6-methyladenosine in nuclear RNA is a major substrate of the obesity-associated FTO. Nat Chem Biol. 2011;7(12):885–7. 22002720 10.1038/nchembio.687 PMC3218240

[17] Visvanathan A, et al. Essential role of METTL3-mediated m(6)A modification in glioma stem-like cells maintenance and radioresistance. Oncogene. 2018;37(4):522–33. 28991227 10.1038/onc.2017.351

[18] Chang YZ, et al. METTL3 enhances the stability of MALAT1 with the assistance of HuR via m^6^A modification and activates NF-kappaB to promote the malignant progression of IDH-wildtype glioma. Cancer Lett. 2021;511:36–46. 33933553 10.1016/j.canlet.2021.04.020

[19] Sun Y, et al. METTL3 promotes chemoresistance in small cell lung cancer by inducing mitophagy. J Exp Clin Cancer Res. 2023;42(1):65. 36932427 10.1186/s13046-023-02638-9 PMC10022264

[20] Shen S, et al. An epitranscriptomic mechanism underlies selective mRNA translation remodelling in melanoma persister cells. Nat Commun. 2019;10(1):5713. 31844050 10.1038/s41467-019-13360-6 PMC6915789

[21] Mitchell K, et al. The evolution of the cancer stem cell state in glioblastoma: emerging insights into the next generation of functional interactions. Neuro Oncol. 2021;23(2):199–213. 33173943 10.1093/neuonc/noaa259 PMC7906055

[22] Bao S, et al. Glioma stem cells promote radioresistance by preferential activation of the DNA damage response. Nature. 2006;444(7120):756–60. 17051156 10.1038/nature05236

[23] Singh SK, et al. Identification of human brain tumour initiating cells. Nature. 2004;432(7015):396–401. 15549107 10.1038/nature03128

[24] Li Y, et al. Lysine methylation promotes NFAT5 activation and determines temozolomide efficacy in glioblastoma. Nat Commun. 2023;14(1):4062. 37429858 10.1038/s41467-023-39845-z PMC10333326

[25] Hu C, et al. Hypoxia improves self-renew and migration of urine-derived stem cells by upregulating autophagy and mitochondrial function through ERK signal pathway. Mitochondrion. 2023;73:1–9. 37678426 10.1016/j.mito.2023.09.001

[26] Liu D, et al. Hypoxia-induced galectin-8 maintains stemness in glioma stem cells via autophagy regulation. Neuro Oncol. 2024;26(5):872–88. 38158714 10.1093/neuonc/noad264 PMC11066898

[27] Huang T, et al. MST4 phosphorylation of ATG4B regulates autophagic Activity, Tumorigenicity, and radioresistance in glioblastoma. Cancer Cell. 2017;32(6):840–e8558. 29232556 10.1016/j.ccell.2017.11.005 PMC5734934

[28] Levy JM, et al. Autophagy Inhibition improves chemosensitivity in BRAF(V600E) brain tumors. Cancer Discov. 2014;4(7):773–80. 24823863 10.1158/2159-8290.CD-14-0049 PMC4090283

[29] Sai K, et al. Induction of cell-cycle arrest and apoptosis in glioblastoma stem-like cells by WP1193, a novel small molecule inhibitor of the JAK2/STAT3 pathway. J Neurooncol. 2012;107(3):487–501. 22249692 10.1007/s11060-011-0786-z

[30] Mei X, et al. Glioblastoma stem cell differentiation into endothelial cells evidenced through live-cell imaging. Neuro Oncol. 2017;19(8):1109–18. 28340100 10.1093/neuonc/nox016 PMC5570159

[31] Chen Z, et al. Disruption of beta-catenin-mediated negative feedback reinforces cAMP-induced neuronal differentiation in glioma stem cells. Cell Death Dis. 2022;13(5):493. 35610201 10.1038/s41419-022-04957-9 PMC9130142

[32] Hu Y, Smyth GK. ELDA: extreme limiting dilution analysis for comparing depleted and enriched populations in stem cell and other assays. J Immunol Methods. 2009;347(1–2):70–8. 19567251 10.1016/j.jim.2009.06.008

[33] Ghandi M, et al. Next-generation characterization of the cancer cell line encyclopedia. Nature. 2019;569(7757):503–8. 31068700 10.1038/s41586-019-1186-3 PMC6697103

[34] Brennan CW, et al. The somatic genomic landscape of glioblastoma. Cell. 2013;155(2):462–77. 24120142 10.1016/j.cell.2013.09.034 PMC3910500

[35] Sun HL, et al. Stabilization of ERK-Phosphorylated METTL3 by USP5 increases m(6)A methylation. Mol Cell. 2020;80(4):633–e6477. 33217317 10.1016/j.molcel.2020.10.026 PMC7720844

[36] Yankova E, et al. Small-molecule inhibition of METTL3 as a strategy against myeloid leukaemia. Nature. 2021;593(7860):597–601. 33902106 10.1038/s41586-021-03536-w PMC7613134

[37] Moscat J, Diaz-Meco MT. Feedback on fat: p62-mTORC1-autophagy connections. Cell. 2011;147(4):724–7. 22078874 10.1016/j.cell.2011.10.021 PMC3290994

[38] Venkataramani V, et al. Glioblastoma hijacks neuronal mechanisms for brain invasion. Cell. 2022;185(16):2899–e291731. 35914528 10.1016/j.cell.2022.06.054

[39] Yu P, et al. TERT accelerates BRAF mutant-induced thyroid cancer dedifferentiation and progression by regulating ribosome biogenesis. Sci Adv. 2023;9(35):eadg7125. 37647391 10.1126/sciadv.adg7125 PMC10468137

[40] Attia AS, et al. Association of BRAF(V600E) mutation with the aggressive behavior of papillary thyroid microcarcinoma: a meta-analysis of 33 studies. Int J Mol Sci. 2022. 10.3390/ijms232415626. 36555268 10.3390/ijms232415626 PMC9779545

[41] Shain AH, Bastian BC. From melanocytes to melanomas. Nat Rev Cancer. 2016;16(6):345–58. 27125352 10.1038/nrc.2016.37

[42] Shain AH, et al. Genomic and transcriptomic analysis reveals incremental disruption of key signaling pathways during melanoma evolution. Cancer Cell. 2018;34(1):45–55. e4. 29990500 10.1016/j.ccell.2018.06.005 PMC6319271

[43] Aldape K, et al. Challenges to curing primary brain tumours. Nat Rev Clin Oncol. 2019;16(8):509–20. 30733593 10.1038/s41571-019-0177-5 PMC6650350

[44] Zhou Z, et al. Mechanism of RNA modification N6-methyladenosine in human cancer. Mol Cancer. 2020;19(1):104. 32513173 10.1186/s12943-020-01216-3 PMC7278081

[45] Li F, et al. Interplay of m(6) A and histone modifications contributes to Temozolomide resistance in glioblastoma. Clin Transl Med. 2021;11(9):e553. 34586728 10.1002/ctm2.553 PMC8441140

[46] Cui Q, et al. m(6)A RNA methylation regulates the Self-Renewal and tumorigenesis of glioblastoma stem cells. Cell Rep. 2017;18(11):2622–34. 28297667 10.1016/j.celrep.2017.02.059 PMC5479356

[47] Xiao F, et al. Positive feedback loop of c-myc/XTP6/NDH2/NF-kappaB to promote malignant progression in glioblastoma. J Exp Clin Cancer Res. 2024;43(1):187. 38965580 10.1186/s13046-024-03109-5 PMC11225266

[48] Luo Y, et al. Long noncoding RNA LINC01606 protects colon cancer cells from ferroptotic cell death and promotes stemness by SCD1-Wnt/beta-catenin-TFE3 feedback loop signalling. Clin Transl Med. 2022;12(4):e752. 35485210 10.1002/ctm2.752 PMC9052012

[49] Lavoie H, et al. BRAF oncogenic mutants evade autoinhibition through a common mechanism. Science. 2025;388(6750):eadp2742. 40440367 10.1126/science.adp2742

[50] Debnath J, Gammoh N, Ryan KM. Autophagy and autophagy-related pathways in cancer. Nat Rev Mol Cell Biol. 2023;24(8):560–75. 36864290 10.1038/s41580-023-00585-z PMC9980873

[51] Meena D, Jha S. Autophagy in glioblastoma: a mechanistic perspective. Int J Cancer. 2024;155(4):605–17. 38716809 10.1002/ijc.34991

[52] Tao Z, et al. Autophagy suppresses self-renewal ability and tumorigenicity of glioma-initiating cells and promotes Notch1 degradation. Cell Death Dis. 2018;9(11):1063. 30337536 10.1038/s41419-018-0957-3 PMC6194143

[53] Mulcahy Levy JM, et al. Autophagy inhibition overcomes multiple mechanisms of resistance to BRAF inhibition in brain tumors. Elife. 2017. 10.7554/eLife.19671. 28094001 10.7554/eLife.19671 PMC5241115

[54] Huang X, et al. S-acylation of p62 promotes p62 droplet recruitment into autophagosomes in mammalian autophagy. Mol Cell. 2023;83(19):3485–e350111. 37802024 10.1016/j.molcel.2023.09.004 PMC10552648

[55] Jiang P, Mizushima N. LC3- and p62-based biochemical methods for the analysis of autophagy progression in mammalian cells. Methods. 2015;75:13–8. 25484342 10.1016/j.ymeth.2014.11.021

[56] Duran A, et al. 62 is a key regulator of nutrient sensing in the mTORC1 pathway. Mol Cell. 2011;44(1):134–46. 21981924 10.1016/j.molcel.2011.06.038 PMC3190169

[57] Wen PY, et al. Dabrafenib plus Trametinib in patients with BRAF(V600E)-mutant low-grade and high-grade glioma (ROAR): a multicentre, open-label, single-arm, phase 2, basket trial. Lancet Oncol. 2022;23(1):53–64. 34838156 10.1016/S1470-2045(21)00578-7

[58] Kaley T, et al. BRAF Inhibition in BRAF(V600)-Mutant gliomas: results from the VE-BASKET study. J Clin Oncol. 2018;36(35):3477–84. 30351999 10.1200/JCO.2018.78.9990 PMC6286161

[59] Schreck KC, et al. Response rate and molecular correlates to Encorafenib and binimetinib in BRAF-V600E mutant High-Grade glioma. Clin Cancer Res. 2024;30(10):2048–56. 38446982 10.1158/1078-0432.CCR-23-3241 PMC11096001

[60] Hartsough E, Shao Y, Aplin AE. Resistance to RAF inhibitors revisited. J Invest Dermatol. 2014;134(2):319–25. 24108405 10.1038/jid.2013.358 PMC3947111

[61] Sun C, et al. Reversible and adaptive resistance to BRAF(V600E) inhibition in melanoma. Nature. 2014;508(7494):118–22. 24670642 10.1038/nature13121

[62] Ullah R, et al. RAF-MEK-ERK pathway in cancer evolution and treatment. Semin Cancer Biol. 2022;85:123–54. 33992782 10.1016/j.semcancer.2021.05.010

