# Supplementary material for: The BRAF V600E/MEK/ERK/METTL3 positive feedback loop regulates autophagy and promotes stemness and invasiveness in glioblastoma via m6A modification
Source: J Exp Clin Cancer Res. 2025 Dec 30;45:28. doi: 10.1186/s13046-025-03623-0 (PMC12853985; doi:10.1186/s13046-025-03623-0)
Supplement: Supplementary file 1 — Supplementary Material 1. [file 13046_2025_3623_MOESM1_ESM.pdf]

Supplementary Figure S1

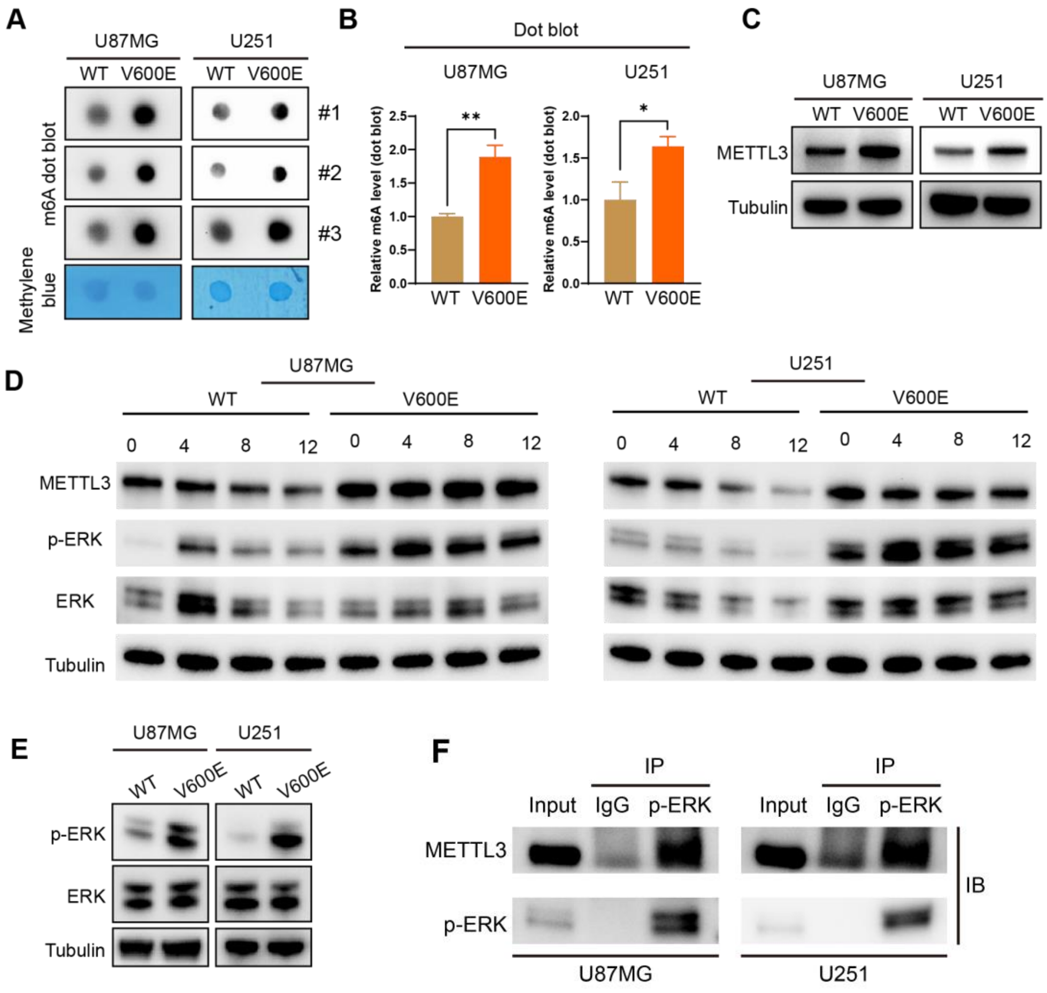

Supplementary Figure S1.

(A-B) m^{6}A dot blot and quantification of global m^{6}A abundance in U87MG and U251 cells with or without BRAF V600E

(C) METTL3 protein expression levels in U87MG and U251 cells with or without BRAF V600E, as analyzed by western blot.

(D) U87MG and U251 cells with or without BRAF V600E were treated with CHX (0, 4, 8, and 12 h). METTL3 protein stability was analyzed by western blot.

(E) p-ERK and ERK protein expression in U87MG and U251 cells with or without BRAF V600E, as analyzed by western blot.

(F) Co-IP analysis of the interaction between p-ERK and METTL3 in U87MG and U251 cells. Cell lysates were immunoprecipitated with anti-p-ERK and immunoblotted with anti-METTL3.

*$p < 0.05$, **$p < 0.01$. ***$p < 0.001$.

Supplementary Figure S2

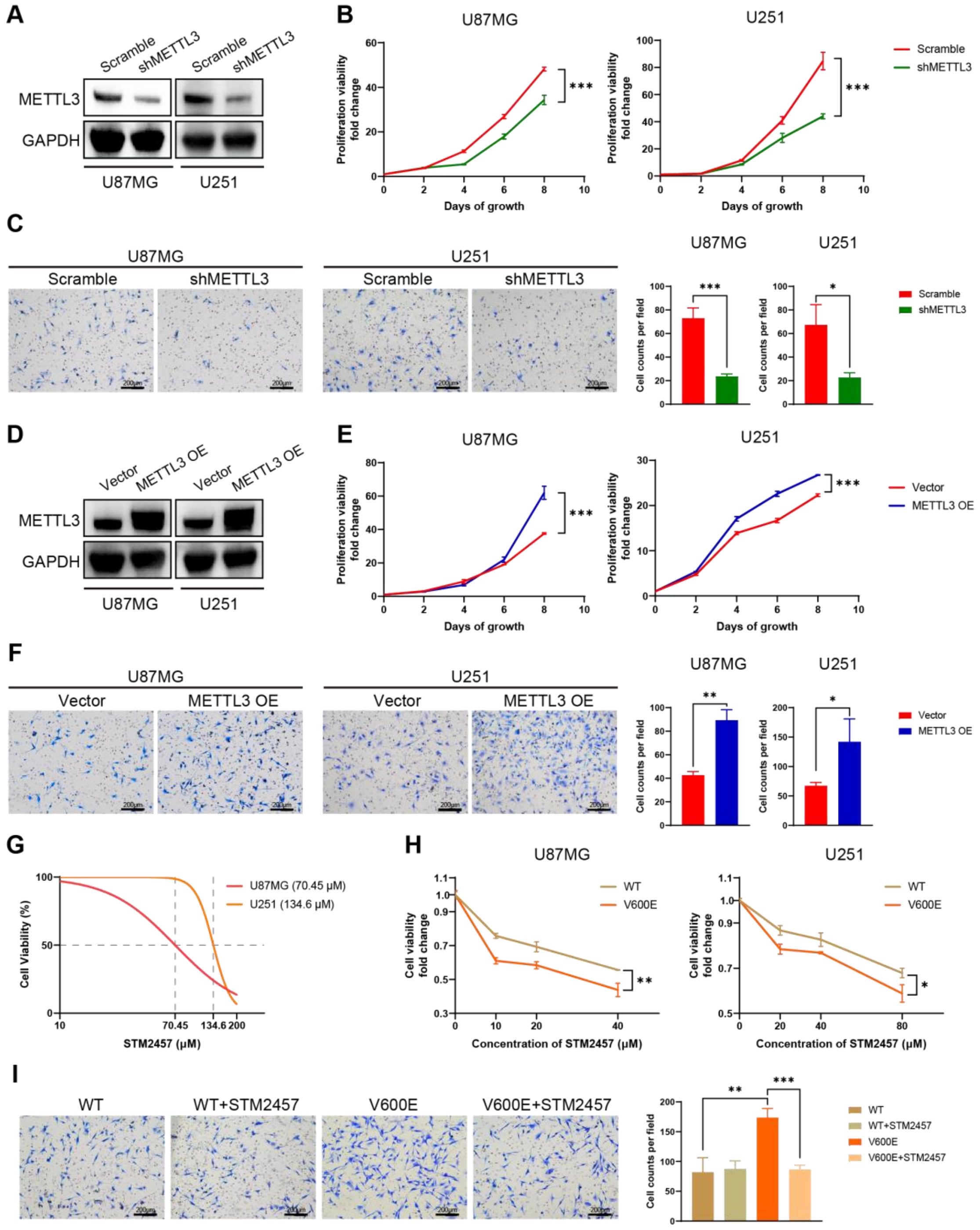

Supplementary Figure S2.

(A) METTL3 expression in METTL3-knockdown U87MG and U251 cells using stable lentiviral transfection, as analyzed by western blot.

(B) CCK-8 assay results showing reduced proliferation viability in U87MG and U251 cells after METTL3 knockdown.

(C) Transwell assays assessing cell migration in METTL3-knockdown U87MG and U251 cells.

Scale bars: 200 μm.

(D) METTL3 expression in METTL3-overexpressing U87MG and U251 cells using stable lentiviral transfection, as analyzed by western blot.

(E) CCK-8 assays showing increased proliferation viability in METTL3-overexpressing U87MG and U251 cells.

(F) Transwell assays assessing cell migration in METTL3-overexpressing U87MG and U251 cells. Scale bars: 200 μm.

(G) Evaluation of IC50 of the METTL3 inhibitor STM2457 in U87MG and U251 cells by CCK-8 assays.

(H) CCK-8 assays showing the sensitivity of U87MG and U251 cells with or without BRAF V600E to STM2457.

(I) Transwell assays assessing the sensitivity of U87MG cells with or without BRAF V600E to STM2457. Scale bars: 200 μm.

WT, BRAF wild type; V600E, BRAF V600E.*$p < 0.05$, **$p < 0.01$. ***$p < 0.001$.

Supplementary Figure S3

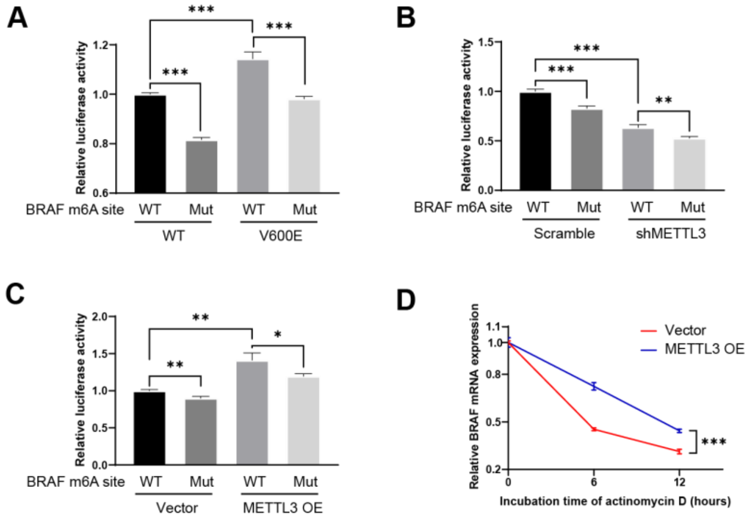

Supplementary Figure S3.

(A–C) Relative luciferase activity of wild-type and mutant BRAF 3′ UTR reporter vectors in U87MG cells: (A) cells with or without BRAF V600E; (B) cells with or without METTL3 knockdown; (C) cells with or without METTL3 overexpression.

(D) Stability of BRAF mRNA in U87MG cells with or without METTL3 overexpression, following transcriptional inhibition by actinomycin D.

*$p < 0.05$, **$p < 0.01$, ***$p < 0.001$.

Supplementary Figure S4

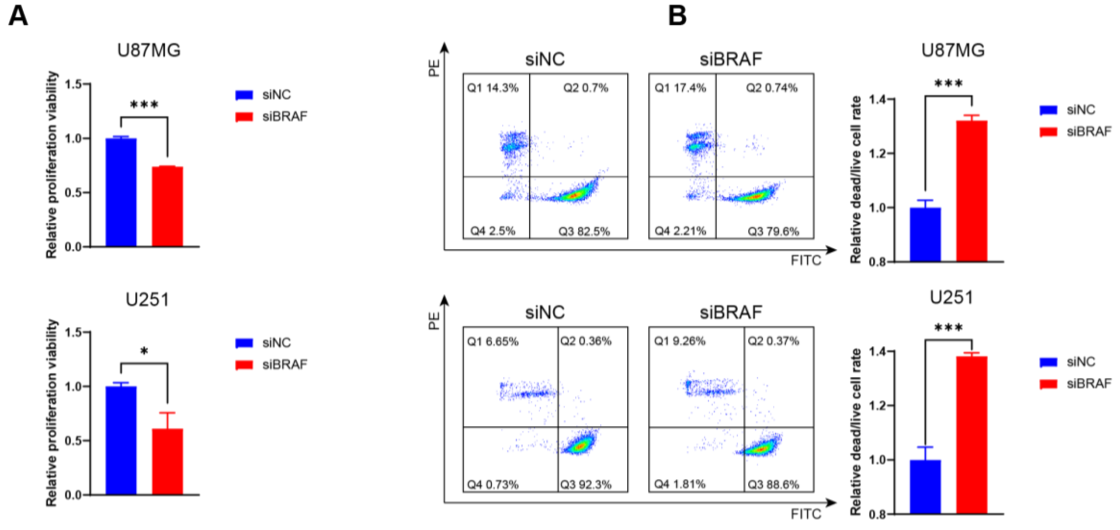

Supplementary Figure S4.

(A) Proliferation viability of U87MG and U251 cells following BRAF knockdown via siRNA, assessed by CCK-8 assay.

(B) Dead/live cell rates in U87MG and U251 cells following BRAF knockdown via siRNA, assessed by flow cytometry.

*$p < 0.05$, ***$p < 0.001$.

Supplementary Figure S5

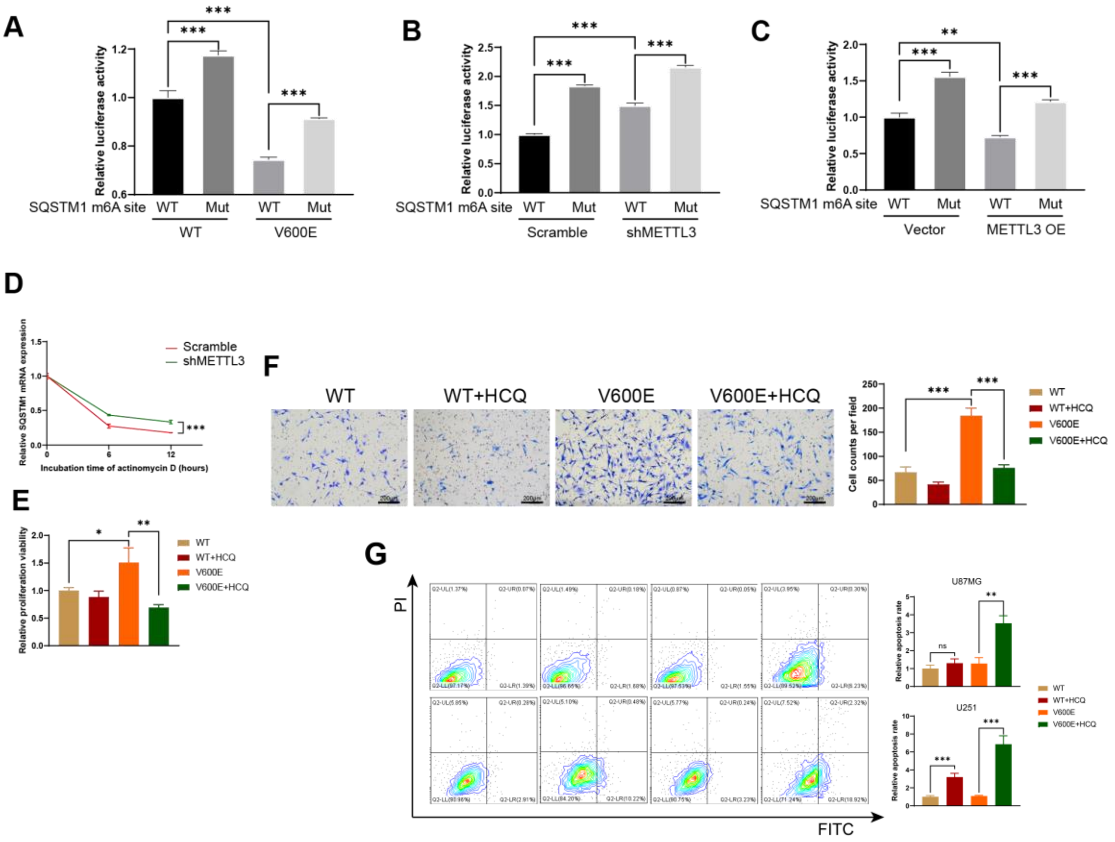

Supplementary Figure S5.

(A–C) Relative luciferase activity of wild-type and mutant SQSTM1 3′ UTR reporter vectors in U87MG cells: (F) cells with or without BRAF V600E; (G) cells with or without METTL3 knockdown; (H) cells with or without METTL3 overexpression.

(D) Stability of SQSTM1 mRNA in U87MG cells with or without METTL3 knockdown, following transcriptional inhibition with actinomycin D.

(E) CCK-8 assay showing the sensitivity of U87MG cells with or without BRAF V600E to HCQ.

(F) Transwell assays assessing the sensitivity of U87MG cells with or without BRAF V600E to HCQ. Scale bars: 200 µm.

(G) Relative apoptosis rates assessing the sensitivity of U87MG and U251 cells with or without BRAF V600E to HCQ, assessed by flow cytometry.

WT, BRAF wild type; V600E, BRAF V600E; HCQ, hydroxychloroquine. ns, no significance, *p < 0.05, **p < 0.01. ***p < 0.001.

Supplementary Figure S6

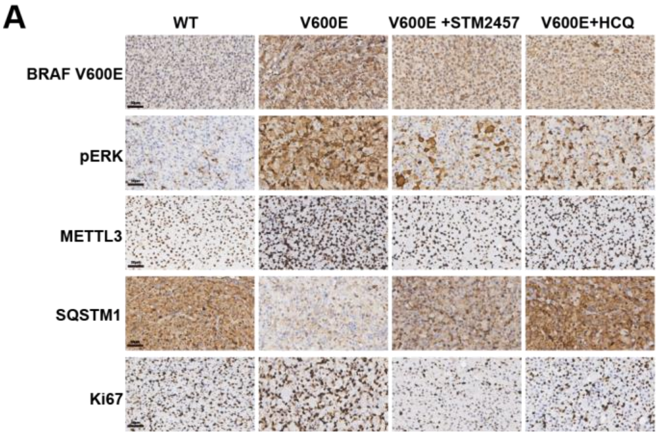

Supplementary Figure S6.

(A) Representative immunohistochemical staining images of brains and GBM tissues from tumor-bearing mice, showing the BRAF V600E, p-ERK, METTL3, SQSTM1, and Ki67 expression in GBM tissues. Scale bars: 50 µm.

Supplementary Table S1. siRNAs and shRNA sequences

Gene	Sequence (5’ to 3’)
BRAF siRNA#1	GCAUCAAUGGAUACCGUUATT
BRAF siRNA#2	CUCCCAAUGUGCAUAUAAATT
METTL3 shRNA	ccggGGTCTGAACTCTTCAGCATCGctcgagCGATGCTGAAGAGTTCAGACCtttttg

Supplementary Table S2. Primer Sequences

primer	Sequence (5’ to 3’)
BRAF-F	TGATGATTGGGAGATTCCTGATGGG
BRAF-R	CTGCTGAGGTGTAGGTGCTGTC
SQSTM1-F	TGATTGAGTCCCTCTCCCAGATGC
SQSTM1-R	CCGCTCCGATGTCATAGTTCTTGG
18S-F	CGAACGTCTGCCCTATCAACTT
18S-R	ACCCGTGGTCACCATGGTA

Supplementary Table S3. Luciferase reporter sequences

luciferase reporter	Sequence (5' to 3')
BRAF-3UTR-WT	AGGGCGGAAAGATCGCCGTGTAATTCTAGATCAGGTAATTACAGT<br>TTATTTCCCACCCTCACATTAATTTTTCCCTATCCTAGATTTACTGC<br>CACCTCACCCCATTTTGGTCTCTGTTCTCTACATCTGTGCCTACTCT<br>GTGAGCTTTGTTGTTTATGCTAGTTTGCAGTGACTTCCAACTCATAC<br>TCCACCAAAACACACGTGGGTTCATCTAGAGTCGGGGCGGCCGGC<br>CGCTTCGAG
BRAF-3UTR-MUT	AGGGCGGAAAGATCGCCGTGTAATTCTAGATCAGGTAATTACAGT<br>TTATTTCCCACCCTCACATTAATTTTTCCCTATCCTAGATTTACTGC<br>CACCTCACCCCATTTTGGTCTCTGTTCTCTACATCTGTGCCTACTCT<br>GTGAGCTTTGTTGTTTATGCTAGTTTGCAGTGACTTCCAACTCATAC<br>TCCACCAAATCACACGTGGGTTCATCTAGAGTCGGGGCGGCCGGC<br>CGCTTCGAG
SQSTM1-3UTR-WT	AGGGCGGAAAGATCGCCGTGTAATTCTAGATCTCCCGCCAGAGGC<br>TGACCCGCGGCTGATTGAGTCCCTCTCCCAGATGCTGTCCATGGGC<br>TTCTCTGATGAAGGCGGCTGGCTCACCAGGCTCCTGCAGACCAAG<br>AACTATGACATCGGAGCGGCTCTGGACACCATCCAGTATTCAAAG<br>CATCCCCCGCCGTTGTCTAGAGTCGGGGCGGCCGGCCGCTTCGAG
SQSTM1-3UTR-MUT	AGGGCGGAAAGATCGCCGTGTAATTCTAGATCTCCCGCCAGAGGC<br>TGACCCGCGGCTGATTGAGTCCCTCTCCCAGATGCTGTCCATGGGC<br>TTCTCTGATGAAGGCGGCTGGCTCACCAGGCTCCTGCAGTCCAAGA<br>ACTATGACATCGGAGCGGCTCTGGACACCATCCAGTATTCAAAGC<br>ATCCCCCGCCGTTGTCTAGAGTCGGGGCGGCCGGCCGCTTCGAG
